# Profiling and annotation of human kidney glomerulus proteome

**DOI:** 10.1186/1477-5956-11-13

**Published:** 2013-04-08

**Authors:** Zenyui Cui, Yutaka Yoshida, Bo Xu, Ying Zhang, Masaaki Nameta, Sameh Magdeldin, Tomoo Makiguchi, Toshikazu Ikoma, Hidehiko Fujinaka, Eishin Yaoita, Tadashi Yamamoto

**Affiliations:** 1 Department of Structural Pathology, Institute of Nephrology, Graduate School of Medical and Dental Sciences, Niigata University, Niigata, Japan; 2 Department of Physiology, Faculty of Veterinary Medicine, Suez Canal University, Ismailia, Egypt; 3 Institute of Clinical Research, Niigata National Hospital, Kashiwazaki, Japan

**Keywords:** Human glomerulus proteome, Human plasma proteome, Human urine proteome, Mouse glomerulus proteome, Cross-reference analysis, Bioinformatics, Biomarkers

## Abstract

**Background:**

The comprehensive analysis of human kidney glomerulus we previously performed using highly purified glomeruli, provided a dataset of 6,686 unique proteins representing 2,966 distinct genes. This dataset, however, contained considerable redundancy resulting from identification criteria under which all the proteins matched with the same set of peptides and its subset were reported as identified proteins. In this study we reanalyzed the raw data using the Mascot search engine and highly stringent criteria in order to select proteins with the highest scores matching peptides with scores exceeding the “Identity Threshold” and one or more unique peptides. This enabled us to exclude proteins with lower scores which only matched the same set of peptides or its subset. This approach provided a high-confidence, non-redundant dataset of identified proteins for extensive profiling, annotation, and comparison with other proteome datasets that can provide biologically relevant knowledge of glomerulus proteome.

**Results:**

Protein identification using the Mascot search engine under highly stringent, computational strategy generated a non-redundant dataset of 1,817 proteins representing 1,478 genes. These proteins were represented by 2-D protein array specifying observed molecular weight and isoelectric point range of identified proteins to demonstrate differences in the observed and calculated physicochemical properties. Characteristics of glomerulus proteome could be illustrated by GO analysis and protein classification. The depth of proteomic analysis was well documented via comparison of the dynamic range of identified proteins with other proteomic analyses of human glomerulus, as well as a high coverage of biologically important pathways. Comparison of glomerulus proteome with human plasma and urine proteomes, provided by comprehensive analysis, suggested the extent and characteristics of proteins contaminated from plasma and excreted into urine, respectively. Among the latter proteins, several were demonstrated to be highly or specifically localized in the glomerulus by cross-reference analysis with the Human Protein Atlas database, and could be biomarker candidates for glomerular injury. Furthermore, comparison of ortholog proteins identified in human and mouse glomeruli suggest some biologically significant differences in glomerulus proteomes between the two species.

**Conclusions:**

A high-confidence, non-redundant dataset of proteins created by comprehensive proteomic analysis could provide a more extensive understanding of human glomerulus proteome and could be useful as a resource for the discovery of biomarkers and disease-relevant proteins.

## Background

The glomerulus is the site of plasma filtration and production of primary urine in the kidney. Not only does the glomerulus play a pivotal role in the ultrafiltration of plasma into urine but it is also the locus of kidney diseases which often progress to chronic renal failure. The diagnosis and treatment of glomerular diseases are now based on clinical manifestations, urinary protein excretion level, and the renal pathology of needle biopsy specimens. The cellular and matrix architecture of the glomeruli of biopsy specimens have been mapped in detail, providing a basis for diagnosis, classification, and clinical treatment decisions. In contrast to the detailed information about morphological changes in the glomerulus, the molecular composition of the glomerulus and how it changes with the progress of the diseases are still obscure. Identification and characterization of biomarkers and proteins relevant to the onset and progress of diseases, therefore, are of high priority, as they would allow better disease classification, detection, and prognosis. Proteomic analysis of the glomerulus could be the most straightforward approach toward discovery of disease-related proteins [1], while most efforts of proteomic analyses have been focused on urine [2,3].

We have previously analyzed glomeruli purified from kidney cortex with no apparent pathological manifestations in order to compile an in-depth profile of the normal human glomerulus proteome as a resource for clinical research [4]. The large-scale shotgun proteomic analysis provided a dataset of 6,686 identified proteins representing 2,966 distinct genes. The dataset, however, contained considerable redundancy: proteins produced by alternative splicing of primary mRNAs from the same gene and proteins produced from gene families containing highly conservative regions of nucleotide or amino acid sequences are included in the dataset. These proteins were generally identified with lower score and arose from both bioinformatics and biological redundancy because all the proteins matched with the same set of peptides or its subsets were reported as identified proteins. Redundancy is unavoidable in peptide-based protein identification in mass spectrometry, i.e., bottom-up or shotgun analysis. Although we could not exclude the actual presence of these proteins, the redundant dataset obviously contained many ambiguously identified proteins as explained above, and could considerably affect profiling and annotation of proteome under analysis.

In the present study, we reanalyzed raw data obtained in our previous, large-scale proteomic analysis to generate a high-confidence, non-redundant dataset of identified proteins by using the Mascot search engine and highly stringent, computational strategy for extensive profiling and annotation of the normal human glomerulus proteome [5]. The identified proteins were represented by 2-D protein array specifying actual molecular weight (Mw) and isoelectric point (pI) range to demonstrate differences in the observed and calculated physicochemical properties, often lost in shotgun analysis. Characteristics of the glomerulus proteome were illustrated by Gene Ontology (GO) analysis and protein classification with the aid of bioinformatics tools. The depth of proteomic analysis was well documented by comparing the dynamic range of identified proteins with other proteomic analyses targeting the glomerulus. Comparison of the glomerulus proteome with comprehensive analyses of the human plasma and human urine proteomes suggested the extent and characteristics of proteins contaminated from plasma and excreted into urine, respectively. Furthermore, a comparison of ortholog proteins identified in human and mouse glomerulus suggested some biologically significant differences between the two species. The extensive profiling and annotation of the human glomerulus proteome were first conducted in this study and could be a useful resource for the discovery of biomarkers and disease-relevant proteins.

## Results and discussion

### Protein identification

In our previous study [4], glomeruli from the kidney cortex of a 68-year-old male patient who had undergone a nephrectomy due to ureter carcinoma were purified to apparent homogeneity by the sieving method. The cortex was histologically normal on the basis of light microscopic observations of PAS- and PAM-stained tissue and no significant deposition of immunoglobulins (IgA, IgG, and IgM) or complement C3 was observed by immunofluorescence microscopy. In the previous study, protein extract from the glomerular preparation was fractionated by two procedures; 1-D SDS-PAGE and 2-D pre-fractionation using solution phase isoelectric focusing (IEF) followed by 1-D-SDS-PAGE. All SDS-PAGE lanes were cut into 15 slices corresponding to a total of 90 slices or fractions, processed by in-gel trypsin digestion, and analyzed by a nanoflow-ESI-ion trap mass spectrometer. Peptides recovered from each gel slice were analyzed in duplicate by two consecutive LC-MS/MS runs followed by two consecutive blank LC-MS/MS runs. The four raw data files generated were merged and identified using the Spectrum Mill search engine (for details, see Additional file 1). In this study, the four raw data files for each of the fractions were converted into Mascot generic files via a Data Analysis software (Agilent) and used for protein identification using the Mascot search engine against the IPI_human protein sequence database (ver. 3.70) to create a dataset of high-confidence, non-redundant identified proteins (see Methods section). In addition, the same raw data files were analyzed by Spectrum Mill using the same version of the IPI_human protein sequence database for convenience of comparison.

Datasets of proteins identified by Spectrum Mill and Mascot are summarized in Table 1 (for details of protein IDs by Mascot, see Additional file 2). There was a significant difference in the number of proteins identified by Spectrum Mill and Mascot, which could be explained by the presence of considerable redundancy in proteins in the Spectrum Mill dataset, as discussed in “Background” section and Additional file 3.

**Table 1:** Summary of proteins identified by Mascot and Spectrum Mill

**A Proteins identified by Spectrum Mill**			
	**Number of identified proteins**		
**Protein pre-fractionation**	**High confidence**^**a**^	**Low confidence**^**b**^	**Total identified proteins**^**c**^
1-D pre-fractionation^d^	2354	1066	3420
2-D pre-fractionation^e^			
Fr. 1 (pH 3–4.6)	1189	708	1897
Fr. 2 (pH 4.6-5.4)	1753	827	2580
Fr. 3 (pH 5.4-6.2)	1423	886	2309
Fr. 4 (pH6.2-7.0)	2228	909	3137
Fr. 5 (pH 7.0-10.0)	1699	841	2540
*Total number*^f^	*8292*	*4171*	*12463*
Total distinct proteins^g^	4322	1875	**6197**
Total distinct genes^h^			**2085**
**B Proteins identified by Mascot**			
1-D pre-fractionation^d^	543	261	804
2-D pre-fractionation^e^			
Fr. 1 (pH 3–4.6)	268	123	391
Fr. 2 (pH 4.6-5.4)	393	178	571
Fr. 3 (pH 5.4-6.2)	370	139	509
Fr. 4 (pH6.2-7.0)	562	171	733
Fr. 5 (pH 7.0-10.0)	416	147	563
*Total number*^f^	*2009*	*758*	*2767*
Total distinct proteins^g^	1340	477	**1817**
Total distinct genes^h^			**1478**

^a^The number of identified proteins with two or more peptide matches counted by using the non-redundant dataset of identified proteins. ^b^The number of identified proteins with one peptide match counted by using non-redundant dataset of identified proteins. ^c^The sum of the number of identified proteins with high confidence and low confidence as defined above. ^d^Glomerular proteins were directly separated into 15 fractions by one-dimensional (1-D) SDS-PAGE. ^e^Glomerular proteins were separated into 5 fractions with different pI ranges by solution-phase IEF in the first dimension and each fraction was further separated into 15 fractions by 1-D SDS-PAGE in the second dimension. ^f^The sum of the numbers of proteins identified in each fraction of 2-D pre-fractionation with high confidence or low-confidence as defined above. ^g^The sum of identified proteins counted by using the non-redundant dataset created by merging all the identified proteins in fractions of 1-D and 2-D pre-fractionation. ^h^Total distinct proteins included proteins derived from the same genes. In order to identify total distinct genes, the same gene symbols dispersed in the dataset were processed in an Excel work sheet to merge into one gene symbol by using the “Remove Duplicate” command.

**Figure 1:**
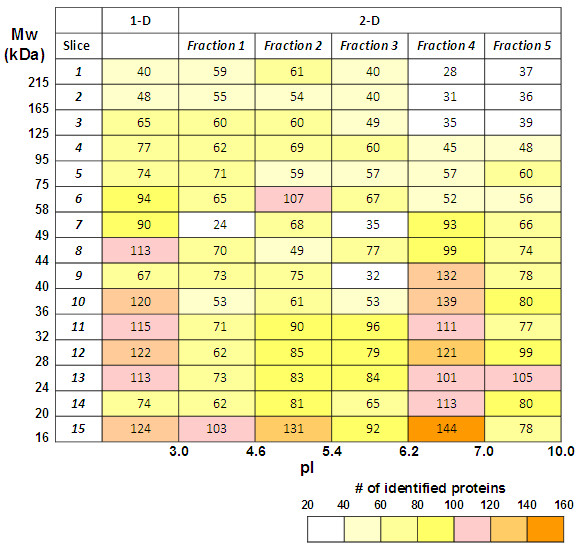
**The number of identified proteins in fractions of 1-D and 2-D pre-fractionation of glomerular proteins.** The number of proteins identified in each of the 90 fractions separated by 1-D (SDS-PAGE) and 2-D pre-fractionation (solution phase isoelectric focusing followed by SDS-PAGE) are shown in 1-D and 2-D protein array format in which each cell or fraction is defined with Mw and pI range. The workflow for the protein identification strategy is depicted in Additional file 1, and details of identified proteins are shown in Additional file 4 and Additional file 5.

The protein identification was conducted with proteins in respective fractions among the 90 fractions obtained by 1-D and 2-D pre-fractionation. Since the respective fractions could be defined by Mw and pI range, proteins identified in these fractions could be provided with the two intrinsic physicochemical properties. We, therefore, compiled the proteins identified in this study into a 1-D protein array consisting of 15 sections defined by Mw range and to a 2-D protein array consisting of 75 sections defined by Mw and pI range (Figure 1, see Additional file 4 and Additional file 5 for details of protein IDs).

**Figure 2:**
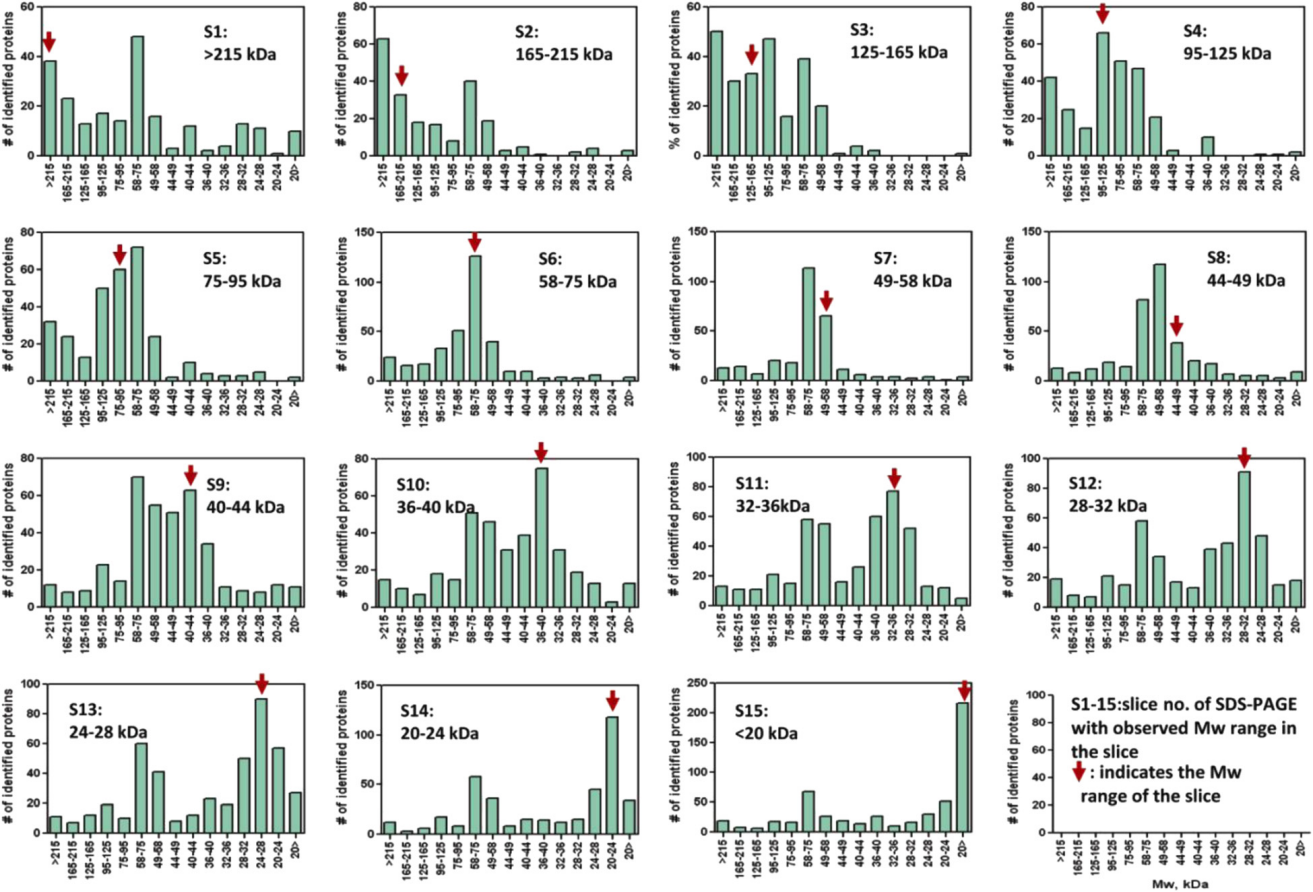
**Distribution of calculated molecular weight of proteins identified in each fraction separated by SDS-PAGE in 2-D prefractionation of glomerular proteins.** Fractions obtained by solution phase isoelectric focusing (fractions 1 to 5) were separated on a 10% SDS-PAGE gel (2-D prefractionation of glomerular proteins), and stained with Coomassie Brilliant blue. Each of the five lanes was cut into 15 slices, and in-gel digested with trypsin for LC-MS/MS analysis. Proteins identified in each of the 5 slices at the same position across the 5 lanes (S1 to S15) were integrated and used to construct a histogram based on calculated (theoretical) Mw as indicated. The red arrow indicates the observed molecular mass range.

**Figure 3:**
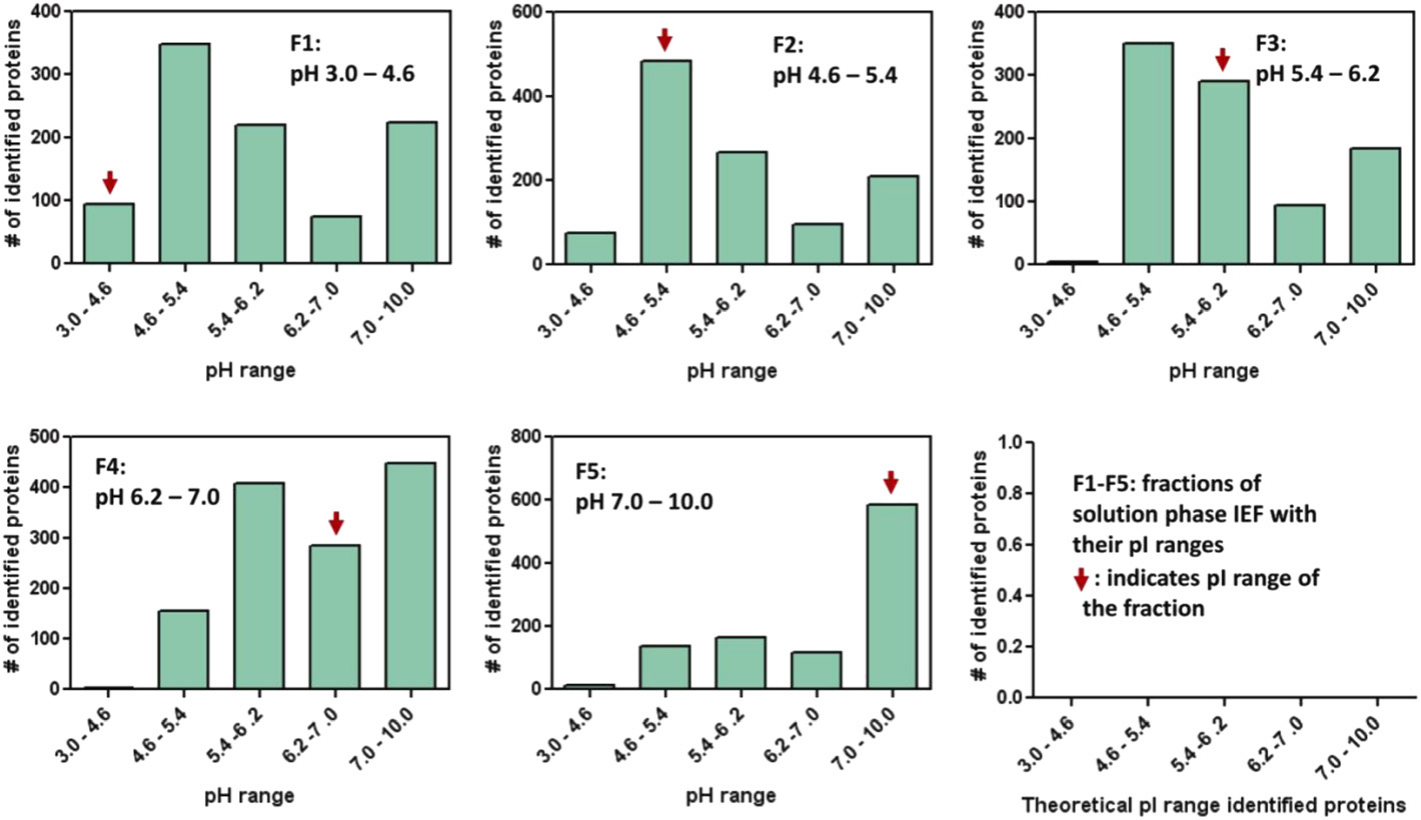
**Distribution of calculated isoelectric point of identified proteins among fractions of solution phase isoelectric focusing.** Proteins fractionated by solution phase IEF (fractions 1 to 5) were separated on a 10% SDS-PAGE gel (2-D prefractionation of glomerular proteins), and stained with Coomassie Brilliant blue. Each of the five lanes was cut into 15 slices, and in-gel digested with trypsin for LC-MS/MS analysis. All the proteins identified in each of the 5 IEF fractions (F1 to F5) were integrated, and used to construct a histogram based on calculated (theoretical) pI as indicated. The red arrow indicates the observed pI range.

Comparison of the 2-D protein array with the distribution of calculated pI and Mw of the identified proteins clearly indicated significant differences between the observed and calculated pI and Mw for the identified proteins. The difference may be illustrated by plotting the calculated Mws of identified proteins in each gel slice of SDS-PAGE gel (Figure 2) and calculated pIs in each fraction of solution phase IEF (Figure 3).

We confirmed successful fractionation with minimal cross-contamination of the solution phase IEF in the first dimension of 2-D pre-fractionation of glomerular proteins [4]. Nevertheless, the difference between observed and calculated pI and Mw was obvious. These observations could possibly be explained by aggregation, degradation or posttranslational modifications (i.e. glycosylation and phosphorylation) of actual proteins in tissue lysate. The solution phase IEF in the first dimension of 2-D pre-fractionation of proteins resulted in extensive condensing of proteins in their pI ranges, which might have contributed to aggregation and/or degradation. The prolonged time required for 2-D pre-fractionation of proteins could also contribute to aggregation as well as degradation of proteins. This result may impose the need for careful consideration on peptide-based targeted proteomics for qualitative and quantitative analysis if protein fractionation is utilized to concentrate target proteins prior to targeted proteomic analysis.

**Figure 4:**
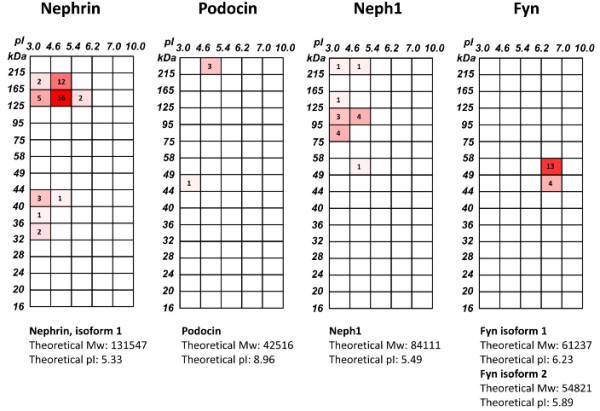
**Location of identification of four key molecules related to the slit diaphragm in fractions of 2-D prefractionation of glomerular proteins.** Distribution of nephrin, podocin, Neph1, and Fyn, known to be key components of the slit diaphragm of podocytes, is shown in the 2-D protein array. Sections, in which these proteins were identified, are highlighted with red colour gradation corresponding to the number of peptides matched to respective proteins. The calculated (theoretical) Mw and pI are indicated below the 2-D protein array for each of the four proteins.

Figure 4 depicts locations in fractions produced by 2-D pre-fractionation of glomerular proteins of the four key molecules related to the slit diaphragm, a critical filtration barrier of plasma proteins in the glomerulus, along with their calculated (theoretical) Mws and pIs. Nephrin, Neph-1 and Fyn were identified mostly in their calculated Mws and pIs, while most podocin was found to be aggregated or associated strongly with large molecular weight proteins and appeared in acidic fractions far from its calculated Mw and pI.

### Characterization of glomerulus proteome using bioinformatics tools

All the identified proteins included in the non-redundant, high-confidence dataset consisting of 1,817 unique proteins representing 1,478 unique genes were subjected to bioinformatics analysis based on the structured vocabulary of Gene Ontology (GO) using a PANTHER analytical tool (ver. 7.0) [6,7]. Subcellular distribution as estimated by analysis with GO Cellular Component vocabulary indicated the highest number of hits to the actin cytoskeleton and a considerably higher number of hits to the intermediate filament cytoskeleton and the extracellular region (a term defining proteins present in the external protective or encapsulating structure outside the plasma membrane including the extracellular matrix) (Additional file 6: Figure S1A). The classification of proteins based on GO Molecular Function vocabulary (Additional file 6: Figure S1B) and Biological Process vocabulary (Additional file 6: Figure S2A) yielded the successful identification of unbiased, widely diverse proteins. Enrichment analysis based on GO Biological Process vocabulary using Cytoscape (version 2.82) with the BinGO plug-in (version 2.42) [8] further illustrated biological processes in which glomerular proteins are significantly enriched compared to products of whole human genes (*p* < 0.001, hypergeometric test followed by multiple testing correction using Benjamin and Hochberg false discovery rate correction) (Additional file 6: Figure S2B).

Under-representation or depletion analysis based on GO Biological Process vocabulary using Cytoscape, as described above, indicated significant depletion of proteins involved in “sensory perception”, “regulation of small GTPase mediated signal transduction”, especially in “regulation of Ras protein signal transduction”, and, most notably, in “regulation of transcription” including “regulation of transcription from RNA polymerase II promoter” (Additional file 7).

PANTHER Protein Class analysis is based on PANTHER Molecular Function ontology, which includes commonly used classes of protein functions, many of which are not covered by GO Molecular Function. PANTHER Protein Class analysis again showed that the highest number of proteins was classified as cytoskeletal proteins (Additional file 6: Figure S3).

### Depth and coverage of proteomic analysis of human kidney glomerulus

We assessed the depth of our comprehensive analysis of the human kidney proteome by comparison with two high-confidence, non-redundant datasets of proteomic analysis of human and mouse glomeruli. The former was the result of analysis of human glomerulus laser-microdissected from frozen sections of biopsy specimens using conventional HPLC in combination with an LTQ-Orbitrap mass spectrometer [9], providing identification of more than 400 proteins from 50 glomerular sections. The latter was the result of analysis of laser-microdissected mouse kidney glomerulus by employing a newly developed nanoflow HPLC on a long, smaller internal diameter column coupled with an LC-MS interface, termed “Replay”. This system allows the direct reanalysis of the injected sample without losing signal intensity [10], providing identification of more than 2,400 proteins from 50 glomerular sections by an LTQ-Orbitrap mass spectrometer.

**Figure 5:**
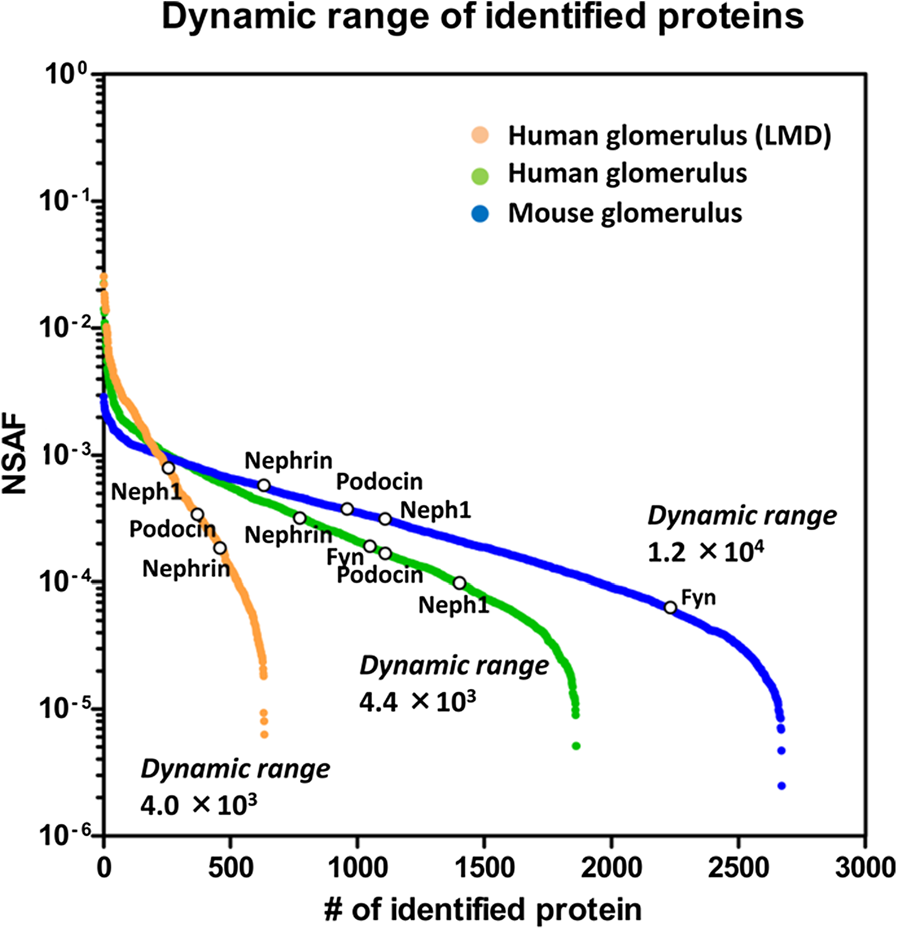
**Comparison of dynamic range of proteomic analyses.** Dynamic ranges of identified proteins achieved by the present study are shown in comparison with those of two proteomic analyses of human (Human glomerulus (LMD)) and mouse glomerulus. The former is the result of analysis of 50 human glomerular sections prepared by laser-microdissection from frozen sections of each of 3 different biopsy specimens using conventional HPLC in combination with an LTQ-Orbitrap mass spectrometer [9]. Four replicate runs were conducted for each of the 3 samples and all the identified proteins were integrated to create a high-confidence, non-redundant dataset of identified proteins (604) under the criteria adopted in this study. The high-confidence, non-redundant dataset of mouse glomerulus proteome was taken from Waanders et al. [10] who analyzed the proteins of 50 glomerular sections laser-microdissected from mouse kidneys using a newly developed nanoflow HPLC. The nanoflow HPLC’s long, smaller internal diameter column coupled with the LC-MS interface “Replay” allowed the reanalysis of the injected sample with high sensitivity, and with an LTQ-Orbitrap mass spectrometer, provided 2,670 proteins. Normalized spectral abundance factor (NSAF), a relative protein abundance index, was calculated according to Paoletti et al. [12]. NSAF is based on the number of peptide matches (spectral counts) divided by protein mass or protein length which roughly correlates with protein concentration in a protein mixture (spectral abundance factor, SAF). To accurately account for run to run variation, individual SAF was normalized by dividing the SAF of a respective protein by the sum of SAFs for all identified proteins to give an NSAF representing relative protein abundance. NSAFs for the four key proteins comprising the slit diaphragm of glomerulus (nephrin, podocin, Neph1 and Fyn) are mapped on each of the abundance curves.

Figure 5 shows a comparison of the dynamic ranges of identified proteins achieved by the three analyses to which the four key proteins related to the slit diaphragm of glomerulus were mapped. As expected, the dynamic range of the proteomic analysis of 50 human glomerular sections was the lowest while those of the proteomic analyses of human glomerulus proteome and the state-of-art analysis of laser-microdissected mouse glomerulus proteome were 1.5, and 3 times higher than the analysis of the 50 human glomerular sections, respectively. It was noted that the number of identified proteins in the middle range of concentrations were much higher in the latter two analyses contributing to the difference in the number of identified proteins. This difference could be attributable to the extensive fractionation of glomerular proteins in the present analysis and to the high-resolution HPLC in combination with the Replay approach of the LC-MS interface employed in the analysis of the 50 mouse glomerular sections. The four slit diaphragm-related proteins, namely nephrin, podocin, Neph1 and Fyn, were identified in the latter two comprehensive analyses whereas among them, Fyn, a non-receptor tyrosine kinase, was lost in the analysis of the 50 human glomerulus sections. DAVID KEGG pathway analysis [11] predicted an over-representation of the actin cytoskeleton regulatory pathway (Additional file 8).

### Comparison of glomerulus proteome with human plasma proteome

**Figure 6:**
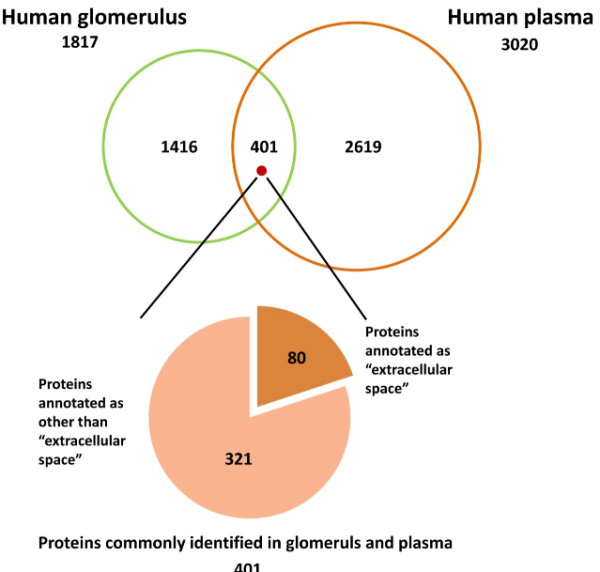
**Comparison of glomerulus proteome with plasma proteome.** Contamination of plasma proteins in the glomerulus proteome was roughly estimated by comparison with a high-confidence, non-redundant dataset of normal human plasma proteome [13]. Among the 401 overlapping proteins between the two proteomes, representing 22.1% of the glomerulus proteome, 40 proteins, annotated as “extracellular space” by GO Cellular Component vocabulary, were selected as plasma proteins to exclude low-abundance, tissue-leakage proteins including intrinsic glomerular proteins. See the text for more details.

Inclusion of blood-derived proteins in glomerulus proteome was unavoidable since the removal of blood from kidney tissue by perfusion with a physiological solution was impossible before the sampling of kidney cortices for the purification of glomeruli. We therefore roughly estimated the extent of blood contamination by comparing glomerulus proteome with normal human plasma proteome consisting of 3,020 non-redundant proteins with two or more peptide matches [13]. Figure 6 depicts the overlap between our glomerulus proteome and the normal human plasma proteome indicating that 401 proteins (22.1% of glomerulus proteome) were commonly identified in both proteomes. Since the plasma proteome contained low-abundant tissue leakage proteins including intrinsic kidney proteins in addition to abundant classical plasma proteins [14], we estimated the extent of contamination of classical plasma proteins in the glomerulus proteome by specifying proteins annotated with “extracellular space” by GO Cellular Component vocabulary, “Extracellular space” is defined as the part of a multicellular organism outside the cell proper, usually taken to be outside the plasma membrane, and occupied by fluid, excluding proteins located in the outmost protective or encapsulating structure of a cell. Among the 401 overlapping proteins, 80 were annotated as “extracellular space” representing plasma proteins (see Additional file 9 for the top 30 plasma proteins identified in glomerulus proteome).

### Comparison of glomerulus proteome with human urine proteome

**Figure 7:**
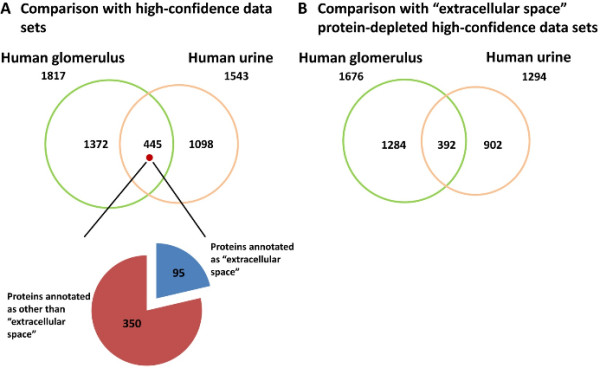
**Comparison of glomerular proteome with urine proteome.** Excretion of glomerular proteins into urine was roughly estimated by comparison with a high-confidence, non-redundant dataset of normal human urine proteome [15]. In Panel **A**, the two proteome datasets were directly compared to estimate overlapped proteins. Among the 445 overlapped proteins representing 24.5% of the glomerular proteome, 95 proteins annotated as “extracellular space” (presumably representing plasma proteins) by GO Cellular Component vocabulary were found. In Panel **B**, the two proteome datasets from which “extracellular space” proteins were depleted were compared to estimate glomerular proteins excreted into urine by removing plasma proteins which were identified in both proteomes.

Most efforts made in proteomics toward the discovery of biomarkers and disease-related proteins of acute kidney injuries (AKI) and chronic kidney diseases (CKD) have been focused on urinary samples because of non-invasiveness collection [2,3], and ease of follow-up study. We compared glomerular proteome with normal human urine proteome consisting of non-redundant high-confidence 1,548 proteins [15]. Figure 7A depicts the overlap between our glomerulus proteome and the normal human urine proteome, indicating that 445 proteins (24.5% of the glomerular proteome) were commonly identified in both proteomes. Figure 7B shows a comparison of the two proteomes deprived of “extracellular space” proteins to remove plasma proteins as discussed above, which were present in both proteomes, again indicating that 23.4% of glomerular proteins were commonly identified in both proteomes. Among the 392 proteins, we looked for proteins originated from the glomerulus by searching in the Human Protein Atlas, an antibody-based, comprehensive proteome database for the systemic exploration of human proteome by profiling cells and tissues using immunohistochemistry [16]. Additional file 10 provides 59 proteins which were shown to be expressed in the glomerulus in the Human Protein Atlas. These proteins may include possible urinary biomarkers for glomerular injury in AKI and CKD, although confirmation by clinical studies is necessary.

### Comparison of glomerulus proteome with mouse glomerulus proteome

Waanders et al. reported a comprehensive proteomic analysis of mouse glomerulus prepared by laser microdissection from frozen tissue sections [10], providing identification of more than 2,400 proteins as described above. In the analysis of mouse glomerulus [10], MS/MS data were processed by MaxQuant and searched against the mouse IPI database (ver. 3.37), using Mascot (version 2.1). Parameter settings for their search were similar to those of our study, except that peptide and fragment mass tolerance were set at 7 ppm and 0.5 Da, respectively, and proteins were considered identified when at least two peptides were identified, and at least one of which was uniquely assignable to the respective sequence to exclude redundant protein hits. The false discovery rate (FDR) at the peptide level was set to 1%. The dataset of mouse glomerulus proteome, therefore, was highly confident, non-redundant, and almost comparable to our study. In order to compare both proteomes and gain insight into differences between the two, first, we constructed an ortholog table of human and mouse genes and converted the mouse gene symbols to corresponding human ortholog gene symbols. The ortholog table of human and mouse genes was constructed based on an “Evola ortholog list” (version 7.5) in the Human-Invitational Database (H-Inv DB) [17]. To construct an Evola ortholog list in the H-Inv DB, a list of human genes was created via the mapping of all human full-length transcripts to genomic sequences using three nucleotide sequence alignment search engines including BLAT, BLAST and est2genome. For other species, gene loci were similarly predicted through the mapping of all transcripts from DDBJ, RefSeq and Ensemble nucleotide sequence databases to genomic sequences. The Evola ortholog list was then generated if exons overlapped between species in genomic alignment for the longest for both the human side and the other species’ side: the pairs were detected as ortholog by computational analysis if they could be aligned with a length of 50% or more of the human amino acid sequence. Using this approach, a pair of ortholog genes in the two species under comparison, which were generated from an ancestor gene by specification, was selected.

**Figure 8:**
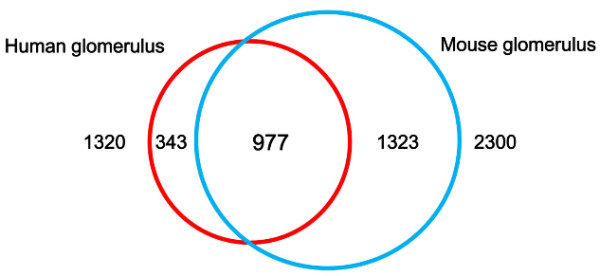
**Comparison of human glomerulus proteome with mouse glomerulus proteome.** The human glomerulus proteome dataset (1,817 proteins, 1,478 genes) was compared with mouse glomerulus proteome [10]. Gene symbols of the mouse dataset were converted to corresponding gene symbols of human ortholog genes by mapping them to an ortholog table of human and mouse genes based on an “Evola ortholog list” (version 7.5) created by the Human Invitational Database (H-Inv DB) [17]. Only the gene symbols of both species that were found in the ortholog table were compared. See the text for more details.

Among 1,478 genes identified in the human glomerulus, 1,320 genes were found in the human-mouse ortholog table while 2,300 genes identified in the mouse glomerulus were found in the table. Figure 8 shows a comparison of ortholog genes identified in both proteomes. The relatively high overlap between the two proteomes (74.0% of genes identified in the human glomerulus proteome) was observed, demonstrating that most of the human genes expressed in glomerulus were also expressed in the mouse glomerulus. The high number of genes uniquely identified in the mouse glomerulus could be attributed to the much higher number of proteins identified in the mouse glomerulus. The relatively high number of genes (343) uniquely identified in the human glomerulus could be explained by the presence of kidney tubular fragments contaminating our glomerulus preparation, identification of human-specific genes such as HLA histocompatibility antigens, or the presence of blood-derived proteins in our glomerular preparation, which are difficult to remove from kidney tissue obtained by nephrectomy. On the other hand, identification of mouse glomerular proteins was conducted with laser-microdissected glomerulus from frozen sections of pre-perfused mouse kidney, allowing the maximal, effective removal of proteins derived from blood and tubules.

The Human Protein Atlas revealed the uniquely identified proteins in the glomerulus (343 genes) to be localized in the kidney (Additional file 11: Table S1). Among them, 162 proteins were found to be localized in the glomerulus in the Human Protein Atlas immunohistochemistry database. We further inspected the proteins expressed in the glomerulus to find proteins expressed highly or specifically in the glomerulus. We found several glomerular proteins which were also confirmed in the literature to be glomerulus-specific proteins including cadherin-13 (gene CDH13) [18], RcPTPNS1 or tyrosine-protein phosphatase non-receptor type substrate 1 (gene SIRPA) [19], isoform 1 of Crumbs homolog 2 (gene CRB2) [20,21], and isoform 1 of secretory phospholipase A2 receptor (gene PLA2R1) [22] (Additional file 11: Table S2). It is interesting to note that PLA2R1 was identified as one of the major target antigens in idiopathic membranous nephropathy [23,24]. Obviously, further experiments are necessary for confirmation, but it might be possible that these proteins are specifically or highly enriched in the human glomerulus in comparison to the mouse glomerulus.

## Conclusions

The raw data produced by our previous comprehensive analysis of human glomerulus proteome were reanalyzed using a high-stringency Mascot search engine to create a high-confidence, non-redundant dataset of identified proteins. This approach provided 1,817 unique proteins representing 1,478 genes and allowed extensive profiling of glomerulus proteome using bioinformatics tools and cross-reference analyses with normal human plasma and urine proteomes, and, in addition, with mouse glomerulus proteome. The considerable difference in the observed and calculated pI and Mw of identified proteins was clearly demonstrated. Analysis based on structured vocabulary of GO indicated the successful identification of a wide unbiased diversity of proteins, and underscored significant enrichment of cytoskeletal and extracellular matrix proteins in the glomerulus proteome. A comparison of the dynamic range of our study with two other proteomic analyses of glomerulus demonstrated the considerable depth of our proteomic analysis in terms of dynamic range and coverage. Comparison of glomerulus proteome with normal human plasma and urine proteomes revealed protein contamination from plasma (amounting to 22.1% of proteins identified in the glomerulus) and excretion into urine (23.4%). A cross-reference analysis with the Human Protein Atlas database indicated the excretion of proteins highly or specifically localized in the glomerulus into urine, suggesting their possible clinical use as urinary biomarkers for glomerular injury. A comparison of ortholog proteins identified in human and mouse glomeruli showed considerable similarity but also suggested some biologically significant differences between species.

## Methods

### LC-tandem mass analysis

The workflow for the preparation of human kidney glomeruli, the strategy for comprehensive analysis, and the LC-MS/MS analysis are provided in Additional file 1. Briefly, 2 mg of proteins extracted from purified glomeruli were either separated on 1-D SDS-PAGE (1-D pre-fractionation) or by 2-D pre-fractionation (solution phase IEF followed by 1-D SDS-PAGE). All the lanes of SDS-PAGE gels were cut into 15 slices (90 slices or fractions in total), and subjected to in-gel digestion to produce tryptic peptides. Two replicate LC-MS/MS runs with samples followed by two consecutive LC-MS/MS runs with blanks (0.3% formic acid) were conducted for each sample. The latter two blank runs were included to retrieve and eliminate carryover peptides from the preceding sample runs.

### Protein identification using Spectrum Mill

We have previously reported identified proteins using Spectrum Mill (version A.03.12.060) as a search engine against the IPI_human protein sequence database (version 3.18) [4]. In this study, we reanalyzed the same raw data set using a new version of Spectrum Mill (version 03.03.081 SR1a) against the IPI_human protein sequence database (version 3.70) for convenience of comparison with proteins identified using the Mascot search engine against the same version of the IPI-human protein sequence database. The identification strategy and criteria for protein identification were similar to those of the previous report [4]. The protein identification done using Spectrum Mill is summarized in Table 1.

### Protein identification using Mascot

For protein identification with the Mascot search engine, the raw data files generated by Spectrum Mill were converted to Mascot generic files (*mgf* files) by using the built-in script of Data Analysis software (Agilent version 6.1) without grouping. The 4 *mgf* files corresponding to each of the fractions (2 sample runs and 2 blank runs) were merged, and searched against the IPI_human protein sequence database (version 3.70) using the Mascot search engine (version 2.2.1). Cystein carbamoidmethylation was set as the fixed modification, and other modifications were set as variable modifications including oxidation of methionine, oxidation of histidine and tryptophan, N-terminal glutamine to pyroglutamate, and N-terminal glutamate to pyroglutamate. Peptide and fragment mass tolerance were set at ±2.5 Da, and at ± 0.7 Da, respectively. A maximum of one missed cleavage was allowed. Proteins matched with at least one unique peptide and with peptides of scores above the “identity threshold” were selected to generate a non-redundant, high-confidence dataset of identified proteins. Selection of matched peptides with scores exceeding the identity threshold was performed by using the same value as the significant threshold in the “Ion score or cut-off” parameter setting. The significant threshold was adjusted to give a false discovery rate of less than 1% (0.25 ± 0.24%), which was calculated on the basis of the number of peptide matches against a decoy database. Protein identification is summarized in Table 1 in comparison with data obtained with Spectrum Mill.

## Abbreviations

AKI: Acute kidney injury; CKD: Chronic kidney diseases; FDR: False discovery rate; GO: Gene Ontology; H-Inv DB: Human-Invitational Database; HPA: Human Protein Atlas; IEF: Isoelectric focusing; mgf: Mascot generic file; Mw: Molecular weight; NSAF: Normalized spectral abundance factor; pI: Isoelectric point.

## Competing interests

All authors have no competing interest.

## Authors’ contributions

ZC designed the study, conducted protein identification with the Mascot search engine, and analyzed identified proteins using bioinformatics tools. YY worked with ZC on all the protein identification procedures, analyses with bioinformatics tools, and cross-reference analyses in the study. BX, YZ and SM worked with ZC on protein identification and helped to analyze using bioinformatics tools. MN participated in the processing of MS raw data for protein identification. TM, TI, HF and EY contributed to the annotation of glomerulus proteome. TY contributed to the overall design of this study and the revision of the manuscript regarding the interpretation of results. All authors read and accepted the final manuscript.

## Supplementary Material

Additional file 1 **Workflow of large-scale proteomic analysis of normal human kidney glomerulus proteome.** The workflow for the large-scale proteomic analysis of normal human kidney glomerulus (*Panel 1.1*) and details of LC-tandem mass analysis (*Panel 1.2*) are provided. Click here for file

Additional file 2 **High-confidence, non-redundant dataset of proteins identified in human kidney glomerulus.** Proteins were identified using Mascot version 2.2.01 searching against the IPI_human protein sequence database (version 3.70), and reported by integrating all the results of 90 fractions prepared by 1-D and 2-D pre-fractionation of 2 mg of protein extracted from a highly purified preparation of normal human kidney glomerulus. See Additional file 1 and the Methods section for details. Click here for file

Additional file 3 **Summary of proteins identified using Spectrum Mill and Mascot.** A graphic view of Table 1 to illustrate the difference in the number of proteins identified by the two search engines is shown in *Panel 3.1*. Venn diagram for comparison of proteins and genes are also shown in *Panel 3.2.* Click here for file

Additional file 4 **Lists of proteins identified in each of the 15 fractions prepared by 1-D pre-fractionation of glomerular proteins.** Proteins identified in each of the 15 slices (fractions) of 1-D SDS-PAGE gel using Mascot are reported in a corresponding worksheet. Click here for file

Additional file 5 **List of proteins identified in 5 fractions prepared by 2-D pre-fractionation of glomerular proteins.** Proteins identified in each of the 75 fractions prepared by 2-D pre-fractionation are compiled in a worksheet. Click here for file

Additional file 6 **Characterization of glomerulus proteome using bioinformatics tools.** All the identified proteins in the non-redundant, high-confidence dataset of glomerulus proteome were analyzed with PANTHER analytical tool (ver. 7.0). Subcellular distribution as analyzed using GO Cellular Component vocabulary (Figure 1A), GO Molecular Function vocabulary (Figure 1B), and GO Biological Process vocabulary (Figure 2A) are shown. In addition, enrichment analysis with GO Biological Process vocabulary and Cytoscape (ver. 2.82) with BinGO plug-in (ver. 2.42) using the results of whole human genes as a background is depicted in Figure 2B. Protein classification analysis using PANTHER Protein Class based on PANTHER Molecular Function ontology is shown in Figure 3. See the text for further details. Click here for file

Additional file 7 **Under-representation analysis of glomerulus proteome.** All the identified proteins of the non-redundant, high-confidence dataset of glomerulus proteome consisting of 1,817 unique proteins representing 1,478 unique genes were analyzed for under-represented or depleted proteins on the basis of GO Biological Process vocabulary with Cytoscape version 2.82 coupled with BinGO plug-in (version 2.42) using the results of whole human genes as a background. Click here for file

Additional file 8 **Glomerular proteins matched in a KEGG “Regulation of Actin Cytoskeleton” pathway.** All the identified proteins of the non-redundant, high-confidence dataset of glomerulus proteome consisting of 1,817 unique proteins representing 1,478 unique genes were analyzed by DAVID KEGG analysis. Matched proteins to components in the “Regulation of Actin Cytoskeleton” are indicated with asterisk. Click here for file

Additional file 9 **An estimate of protein contamination from blood into glomerulus proteome by comparison of glomerulus proteome with plasma proteome.** All the identified proteins of the non-redundant, high-confidence dataset of glomerulus proteome consisting of 1,817 unique proteins representing 1,478 unique genes were compared with the high-confidence, non-redundant dataset of normal human plasma proteome [States DJ et al., *Nat. Biotech.*, 2006, 24, 333–338]. Among the 401 overlapping proteins, proteins annotated as “extracellular space” by GO Cellular Component vocabulary were selected as representing plasma proteins. The top 30 plasma proteins are listed in order according to the number of peptide matches. Click here for file

Additional file 10 **Proteins expressed in the glomerulus excreted into urine.** Among proteins commonly identified in both glomerulus and urine proteome [Adachi et al., *Genome Biol*, 2006, 7, R80], the proteins that were confirmed to be expressed in the glomerulus by immunohistochemistry (Human Protein Atlas) were selected and summarized in this file. Click here for file

Additional file 11 **The proteins that might be specifically or abundantly expressed in human glomerulus compared with mouse glomerulus.** The human glomerular dataset was compared with the mouse glomerular dataset [Waanders et al., *Proc. Natl. Acad. Sci. USA*, 2009, 106, 18902–18907]. Gene symbols of the mouse dataset were converted to corresponding gene symbols of human ortholog genes by mapping them to an ortholog table of human and mouse genes based on an “Evola ortholog list” (version 7.5) created by the Human Invitational database (H-Inv DB). Proteins-corresponding genes uniquely identified in the human dataset were examined for their cellular localization by searching in the Human Protein Atlas (version 9.0). Click here for file
