# Supplementary material for: Profiling and annotation of human kidney glomerulus proteome
Source: Proteome Sci. 2013 Apr 8;11:13. doi: 10.1186/1477-5956-11-13 (PMC3639854; doi:10.1186/1477-5956-11-13)
Supplement: Additional file 1 — Workflow of large-scale proteomic analysis of normal human kidney glomerulus proteome. The workflow for the large-scale proteomic analysis of normal human kidney glomerulus (Panel 1.1) and details of LC-tandem mass analysis (Panel 1.2) are provided. [file 1477-5956-11-13-S1.ppt]

## Slide 1
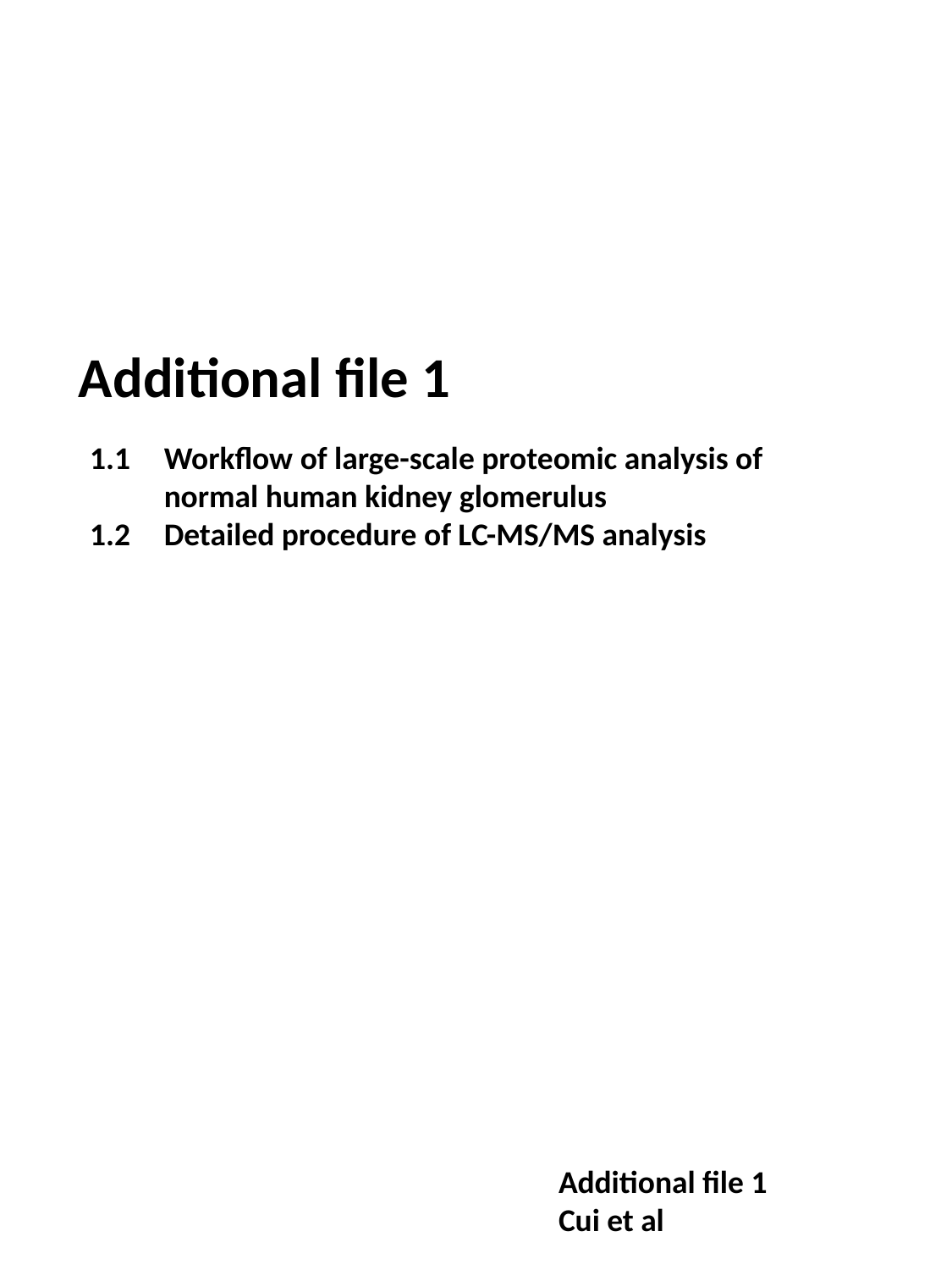

Additional file 1
1.1	Workflow of large-scale proteomic analysis of normal human kidney glomerulus
1.2	Detailed procedure of LC-MS/MS analysis
Additional file 1
Cui et al

## Slide 2
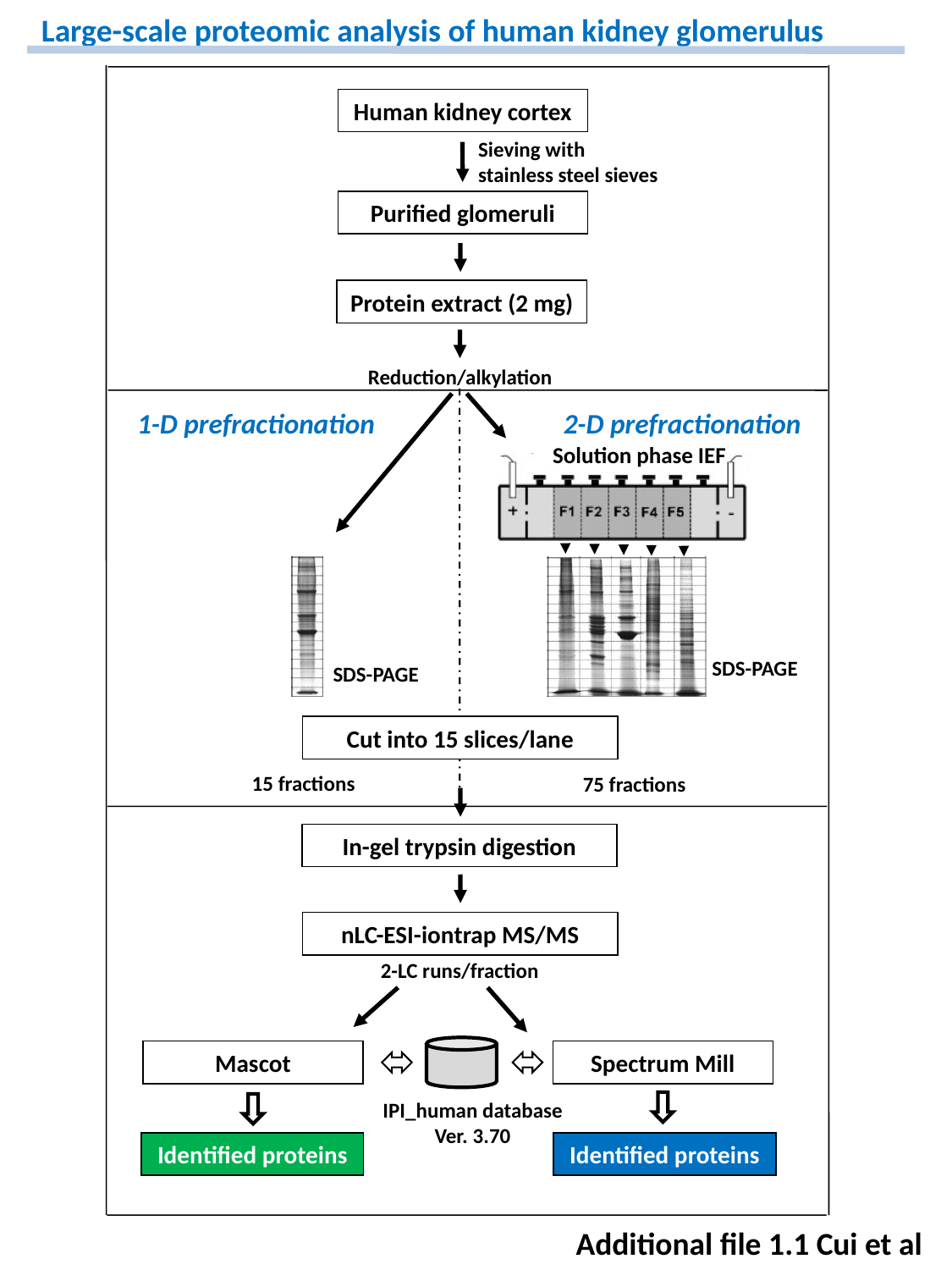

Large-scale proteomic analysis of human kidney glomerulus
Human kidney cortex
Sieving with stainless steel sieves
Purified glomeruli
Protein extract (2 mg)
Reduction/alkylation
1-D prefractionation
2-D prefractionation
Solution phase IEF
SDS-PAGE
SDS-PAGE
Cut into 15 slices/lane
15 fractions
75 fractions
In-gel trypsin digestion
nLC-ESI-iontrap MS/MS
2-LC runs/fraction
Mascot
Spectrum Mill
IPI_human database
Ver. 3.70
Identified proteins
Identified proteins
Additional file 1.1 Cui et al

## Slide 3
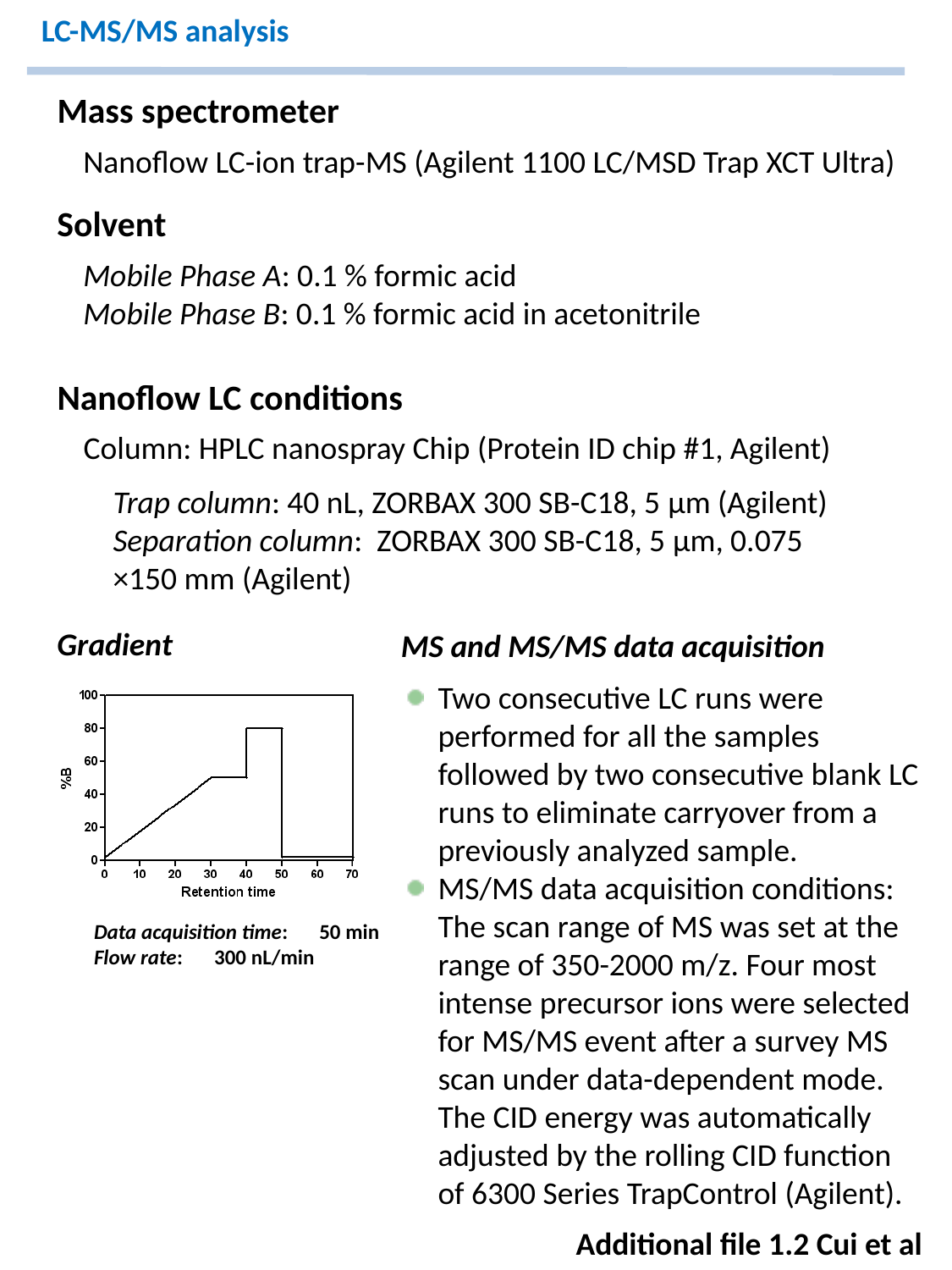

LC-MS/MS analysis
Mass spectrometer
Nanoflow LC-ion trap-MS (Agilent 1100 LC/MSD Trap XCT Ultra)
Solvent
Mobile Phase A: 0.1 % formic acid
Mobile Phase B: 0.1 % formic acid in acetonitrile
Nanoflow LC conditions
Column: HPLC nanospray Chip (Protein ID chip #1, Agilent)
Trap column: 40 nL, ZORBAX 300 SB-C18, 5 μm (Agilent)
Separation column: ZORBAX 300 SB-C18, 5 μm, 0.075 ×150 mm (Agilent)
Gradient
MS and MS/MS data acquisition
Two consecutive LC runs were performed for all the samples followed by two consecutive blank LC runs to eliminate carryover from a previously analyzed sample.
MS/MS data acquisition conditions:
The scan range of MS was set at the range of 350-2000 m/z. Four most intense precursor ions were selected for MS/MS event after a survey MS scan under data-dependent mode. The CID energy was automatically adjusted by the rolling CID function of 6300 Series TrapControl (Agilent).
Data acquisition time:　50 min
Flow rate:　300 nL/min
Additional file 1.2 Cui et al
