# Supplementary material for: Profiling and annotation of human kidney glomerulus proteome
Source: Proteome Sci. 2013 Apr 8;11:13. doi: 10.1186/1477-5956-11-13 (PMC3639854; doi:10.1186/1477-5956-11-13)
Supplement: Additional file 3 — Summary of proteins identified using Spectrum Mill and Mascot. A graphic view of Table 1 to illustrate the difference in the number of proteins identified by the two search engines is shown in Panel 3.1. Venn diagram for comparison of proteins and genes are also shown in Panel 3.2. [file 1477-5956-11-13-S3.ppt]

## Slide 1
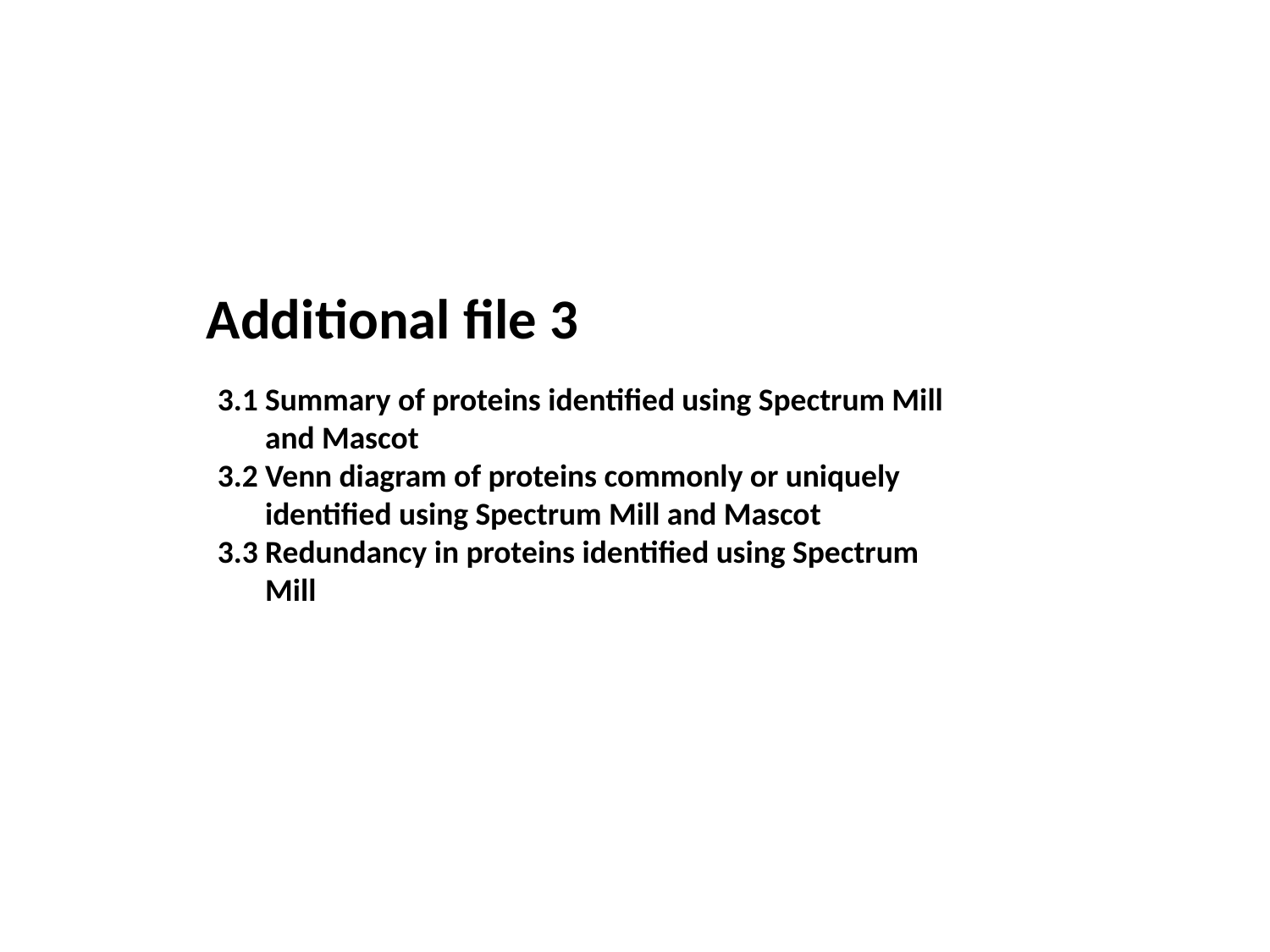

Additional file 3
3.1	Summary of proteins identified using Spectrum Mill and Mascot
3.2	Venn diagram of proteins commonly or uniquely identified using Spectrum Mill and Mascot
3.3	Redundancy in proteins identified using Spectrum Mill

## Slide 2
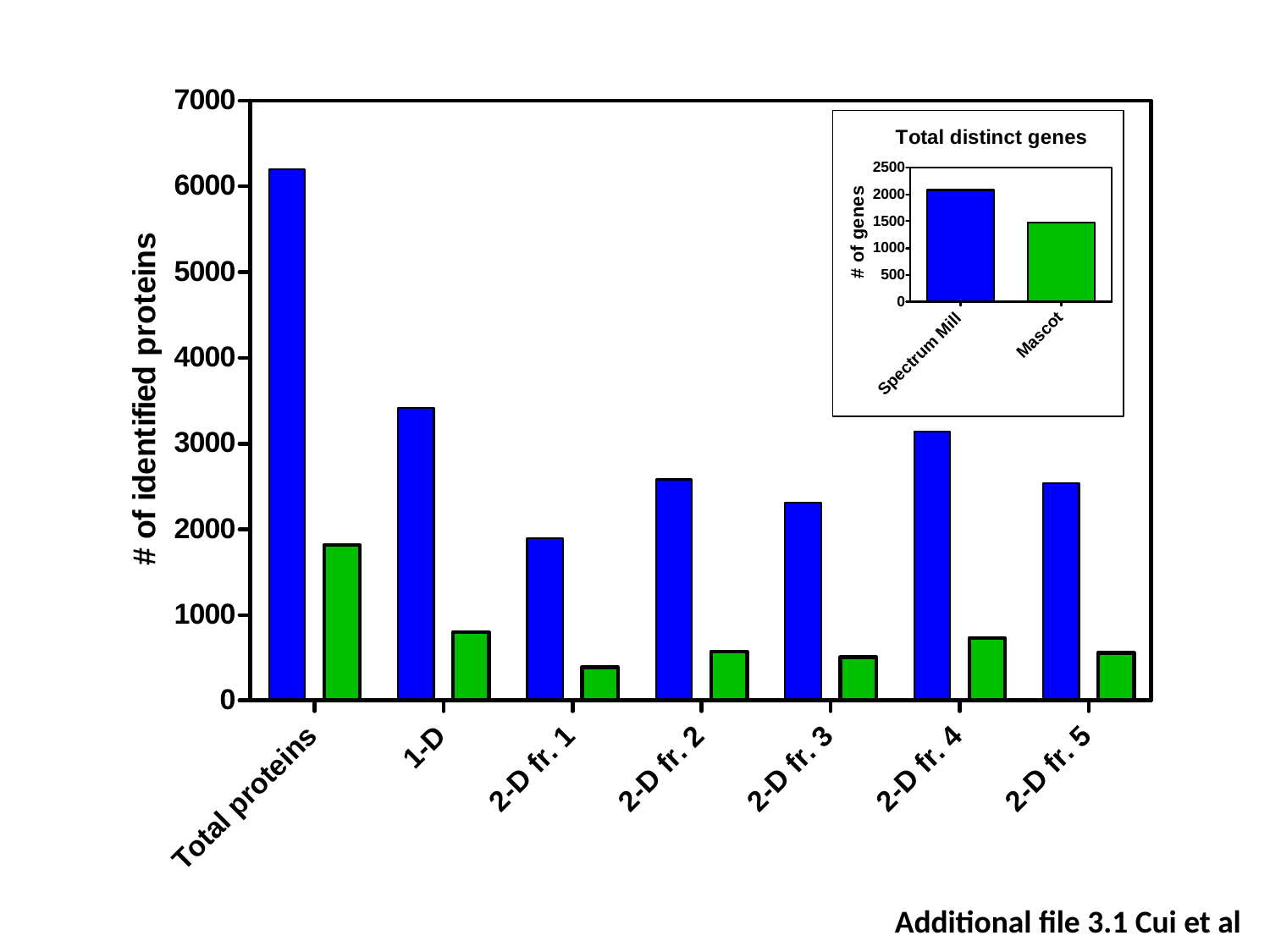

Additional file 3.1 Cui et al

## Slide 3
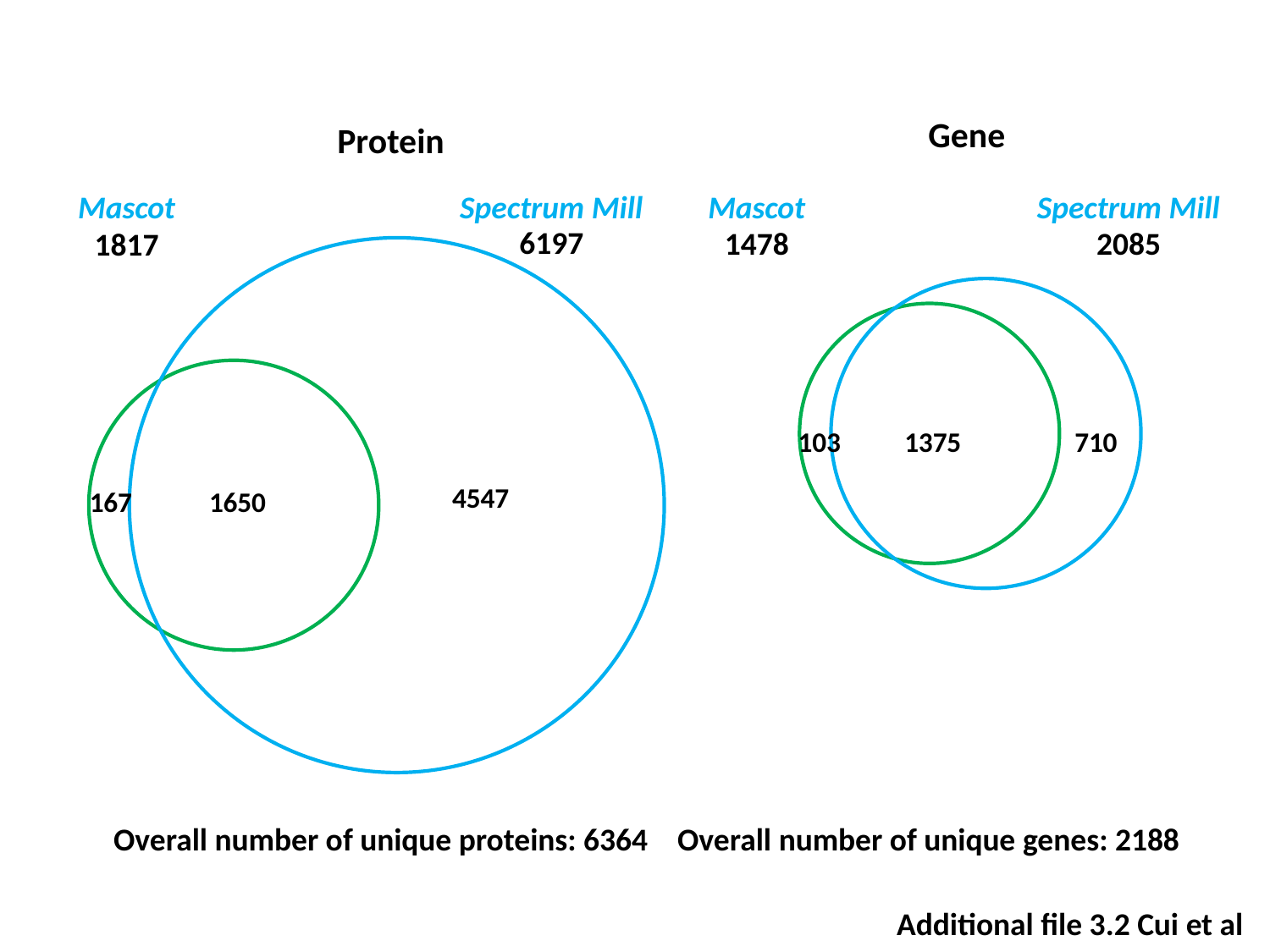

Gene
Protein
Mascot
Spectrum Mill
Mascot
Spectrum Mill
6197
1478
2085
1817
4547
167
1650
103
1375
710
Overall number of unique proteins: 6364
Overall number of unique genes: 2188
Additional file 3.2 Cui et al

## Slide 4
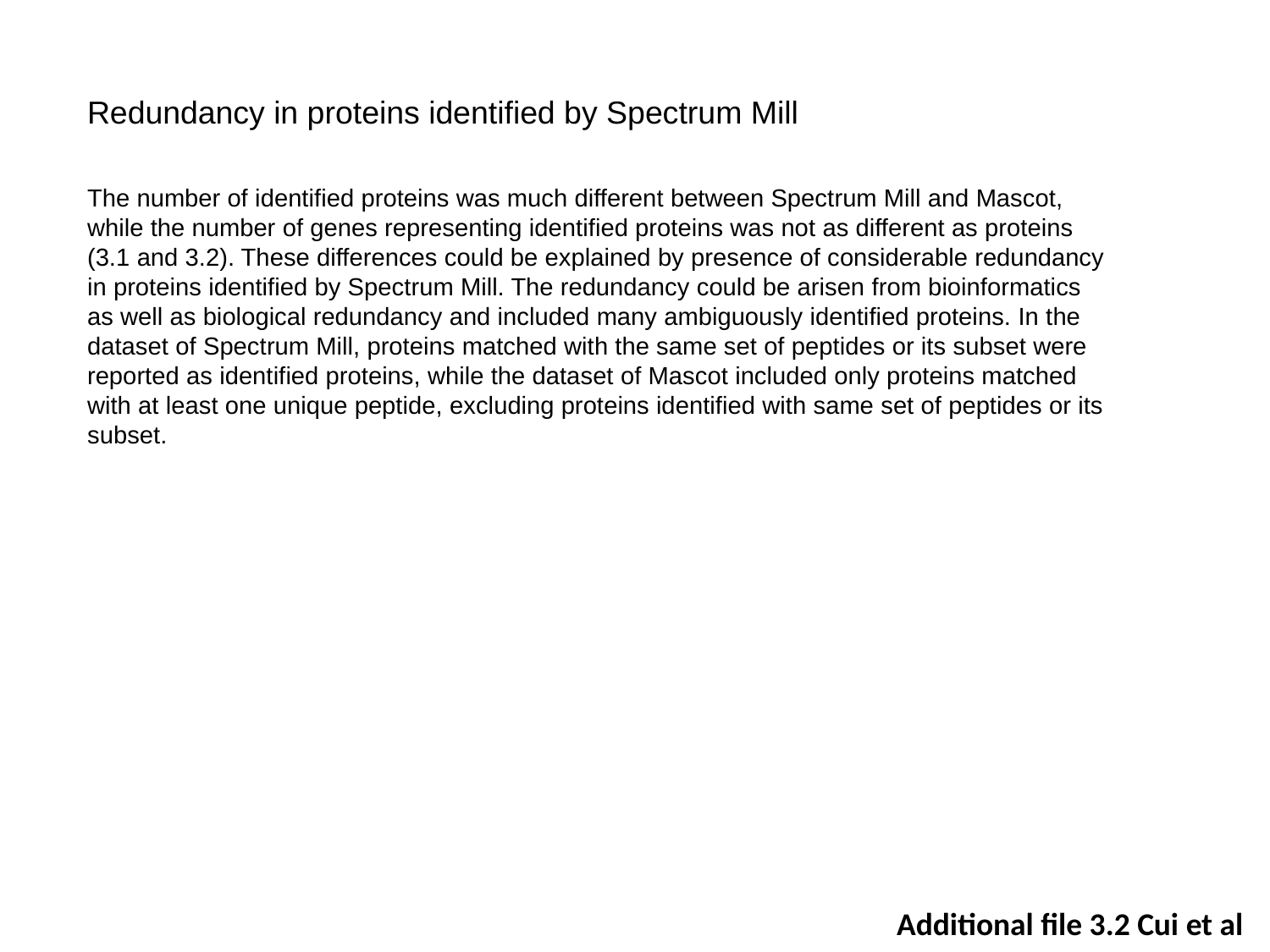

Redundancy in proteins identified by Spectrum Mill
The number of identified proteins was much different between Spectrum Mill and Mascot, while the number of genes representing identified proteins was not as different as proteins (3.1 and 3.2). These differences could be explained by presence of considerable redundancy in proteins identified by Spectrum Mill. The redundancy could be arisen from bioinformatics as well as biological redundancy and included many ambiguously identified proteins. In the dataset of Spectrum Mill, proteins matched with the same set of peptides or its subset were reported as identified proteins, while the dataset of Mascot included only proteins matched with at least one unique peptide, excluding proteins identified with same set of peptides or its subset.
Additional file 3.2 Cui et al
