# Supplementary material for: Profiling and annotation of human kidney glomerulus proteome
Source: Proteome Sci. 2013 Apr 8;11:13. doi: 10.1186/1477-5956-11-13 (PMC3639854; doi:10.1186/1477-5956-11-13)
Supplement: Additional file 6 — Characterization of glomerulus proteome using bioinformatics tools. All the identified proteins in the non-redundant, high-confidence dataset of glomerulus proteome were analyzed with PANTHER analytical tool (ver. 7.0). Subcellular distribution as analyzed using GO Cellular Component vocabulary (Figure 1A), GO Molecular Function vocabulary (Figure 1B), and GO Biological Process vocabulary (Figure 2A) are shown. In addition, enrichment analysis with GO Biological Process vocabulary and Cytoscape (ver. 2.82) with BinGO plug-in (ver. 2.42) using the results of whole human genes as a background is depicted in Figure 2B. Protein classification analysis using PANTHER Protein Class based on PANTHER Molecular Function ontology is shown in Figure 3. See the text for further details. [file 1477-5956-11-13-S6.ppt]

## Slide 1
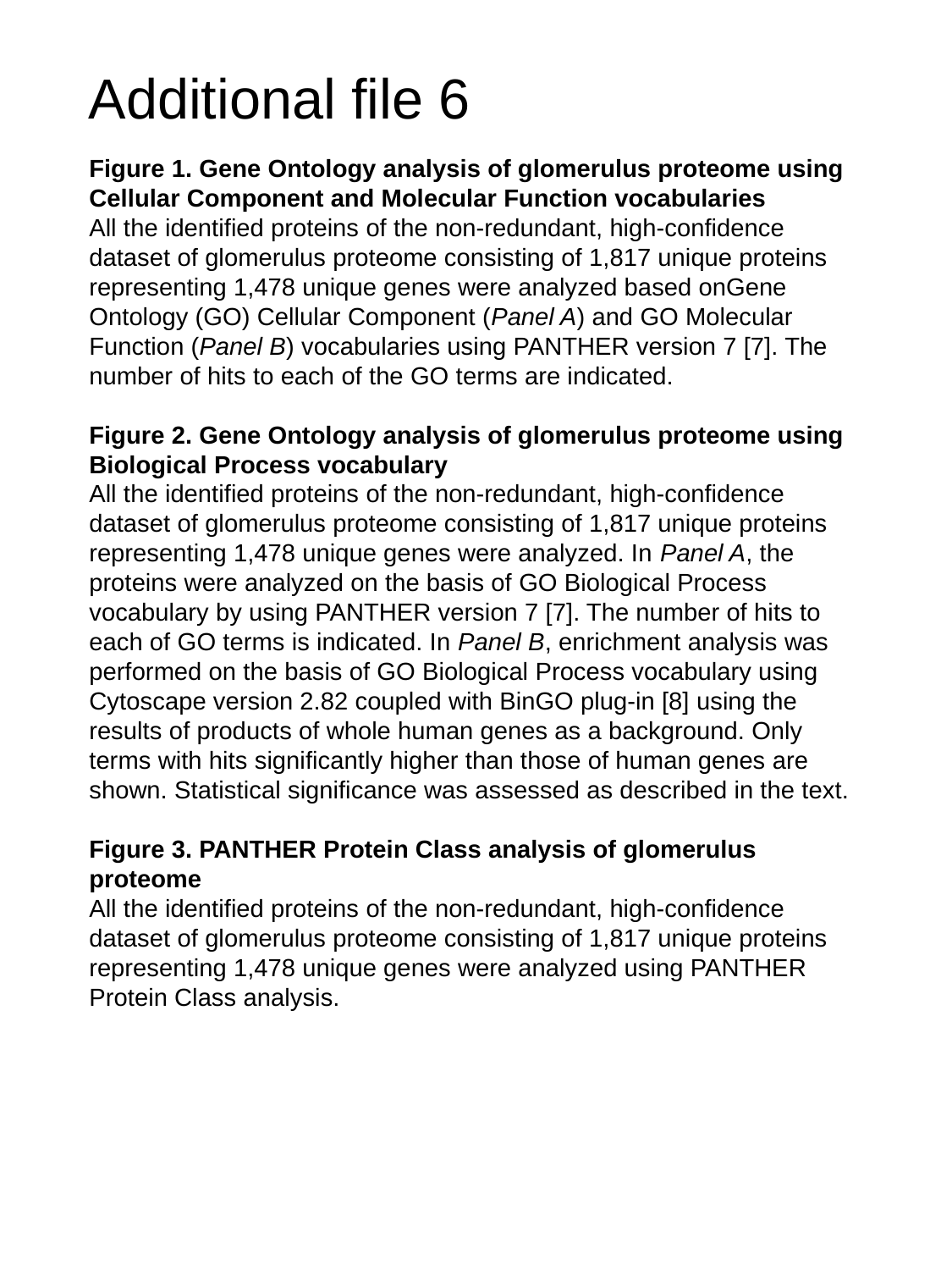

Additional file 6
Figure 1. Gene Ontology analysis of glomerulus proteome using Cellular Component and Molecular Function vocabularies
All the identified proteins of the non-redundant, high-confidence dataset of glomerulus proteome consisting of 1,817 unique proteins representing 1,478 unique genes were analyzed based onGene Ontology (GO) Cellular Component (Panel A) and GO Molecular Function (Panel B) vocabularies using PANTHER version 7 [7]. The number of hits to each of the GO terms are indicated.
Figure 2. Gene Ontology analysis of glomerulus proteome using Biological Process vocabulary
All the identified proteins of the non-redundant, high-confidence dataset of glomerulus proteome consisting of 1,817 unique proteins representing 1,478 unique genes were analyzed. In Panel A, the proteins were analyzed on the basis of GO Biological Process vocabulary by using PANTHER version 7 [7]. The number of hits to each of GO terms is indicated. In Panel B, enrichment analysis was performed on the basis of GO Biological Process vocabulary using Cytoscape version 2.82 coupled with BinGO plug-in [8] using the results of products of whole human genes as a background. Only terms with hits significantly higher than those of human genes are shown. Statistical significance was assessed as described in the text.
Figure 3. PANTHER Protein Class analysis of glomerulus proteome
All the identified proteins of the non-redundant, high-confidence dataset of glomerulus proteome consisting of 1,817 unique proteins representing 1,478 unique genes were analyzed using PANTHER Protein Class analysis.

## Slide 2
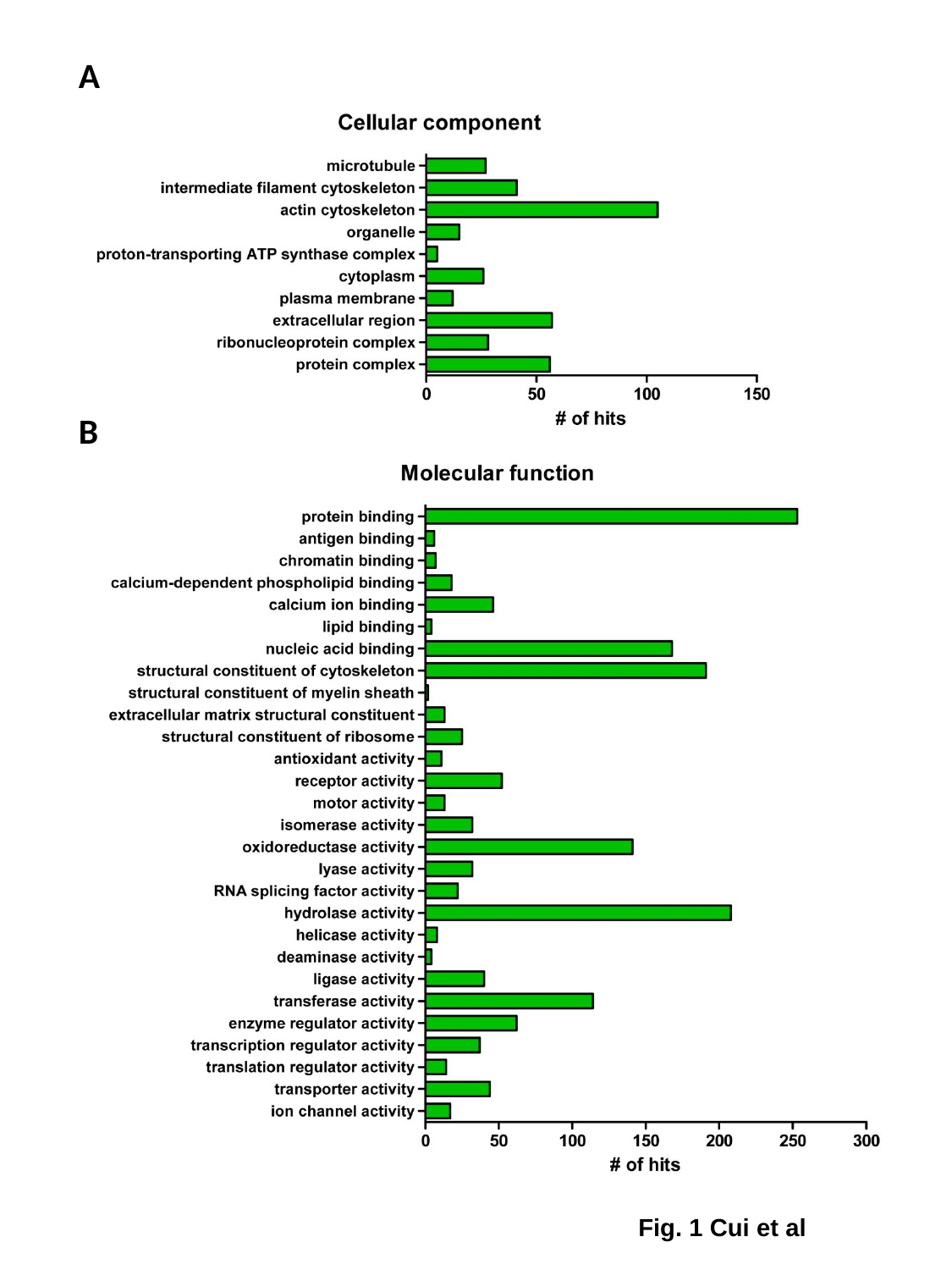

A
B
Fig. 1 Cui et al

## Slide 3
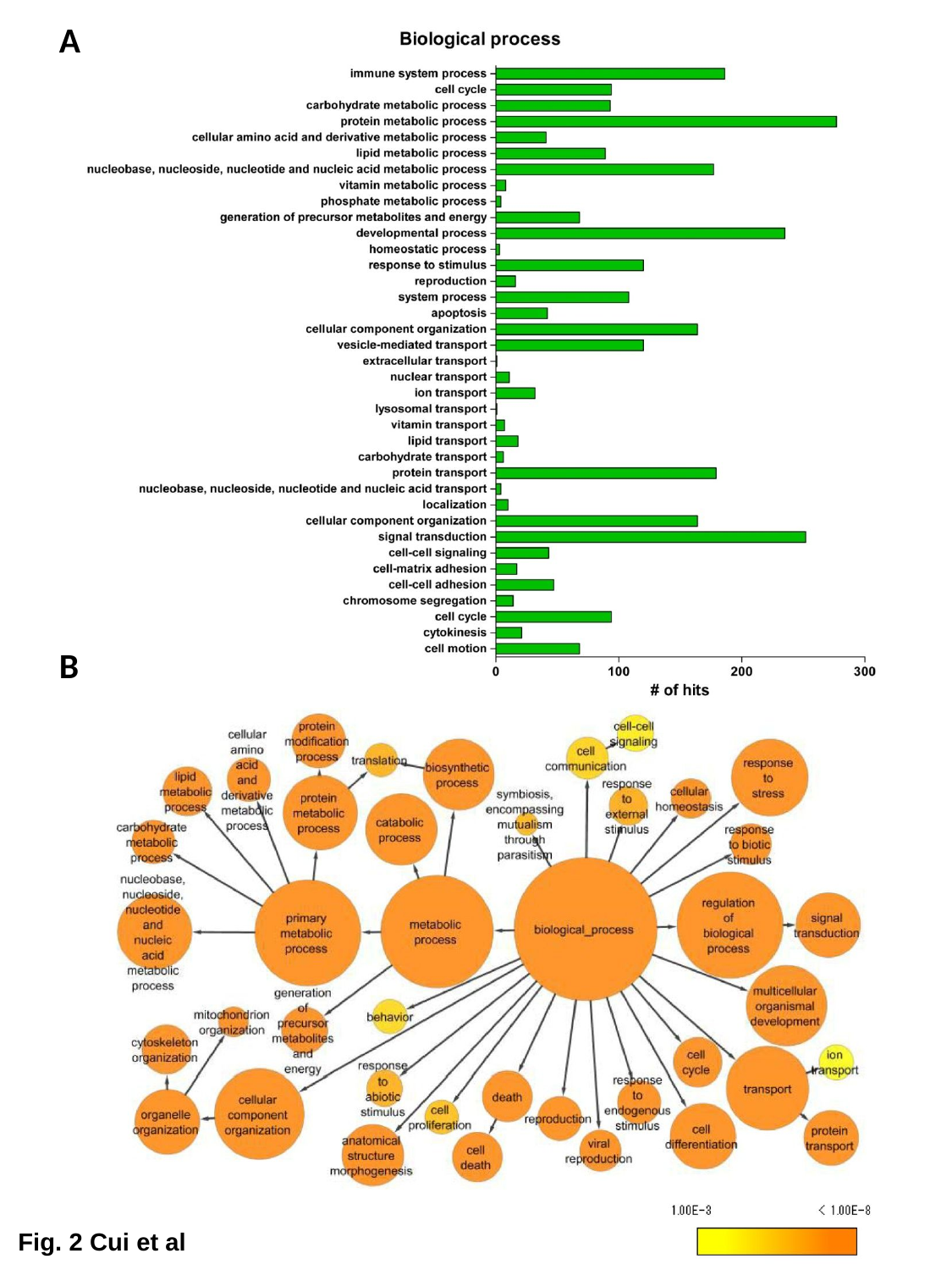

A
B
Fig. 2 Cui et al

## Slide 4
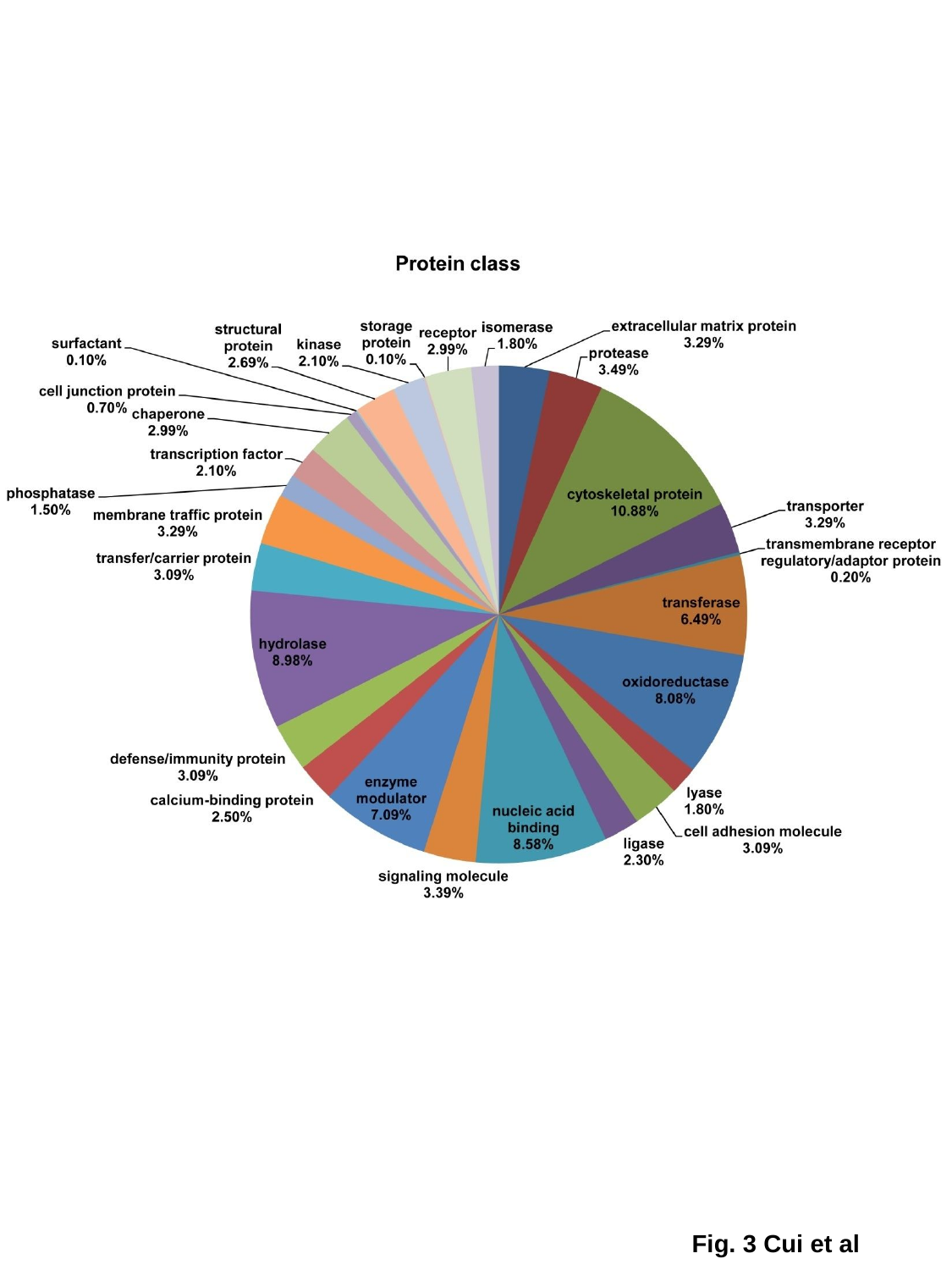

Fig. 3 Cui et al
