# Supplementary material for: Profiling and annotation of human kidney glomerulus proteome
Source: Proteome Sci. 2013 Apr 8;11:13. doi: 10.1186/1477-5956-11-13 (PMC3639854; doi:10.1186/1477-5956-11-13)
Supplement: Additional file 7 — Under-representation analysis of glomerulus proteome. All the identified proteins of the non-redundant, high-confidence dataset of glomerulus proteome consisting of 1,817 unique proteins representing 1,478 unique genes were analyzed for under-represented or depleted proteins on the basis of GO Biological Process vocabulary with Cytoscape version 2.82 coupled with BinGO plug-in (version 2.42) using the results of whole human genes as a background. [file 1477-5956-11-13-S7.ppt]

## Slide 1
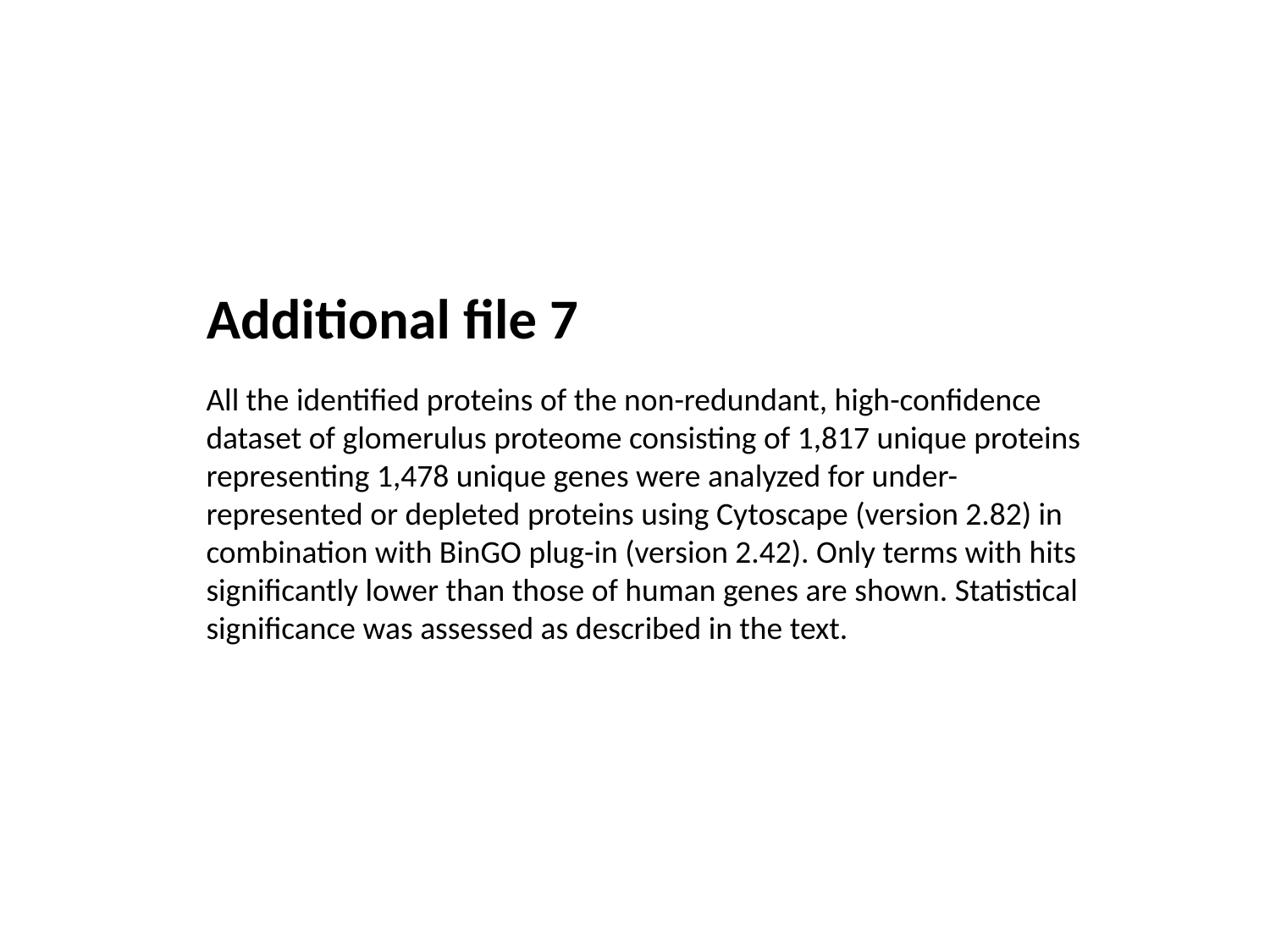

Additional file 7
All the identified proteins of the non-redundant, high-confidence dataset of glomerulus proteome consisting of 1,817 unique proteins representing 1,478 unique genes were analyzed for under-represented or depleted proteins using Cytoscape (version 2.82) in combination with BinGO plug-in (version 2.42). Only terms with hits significantly lower than those of human genes are shown. Statistical significance was assessed as described in the text.

## Slide 2
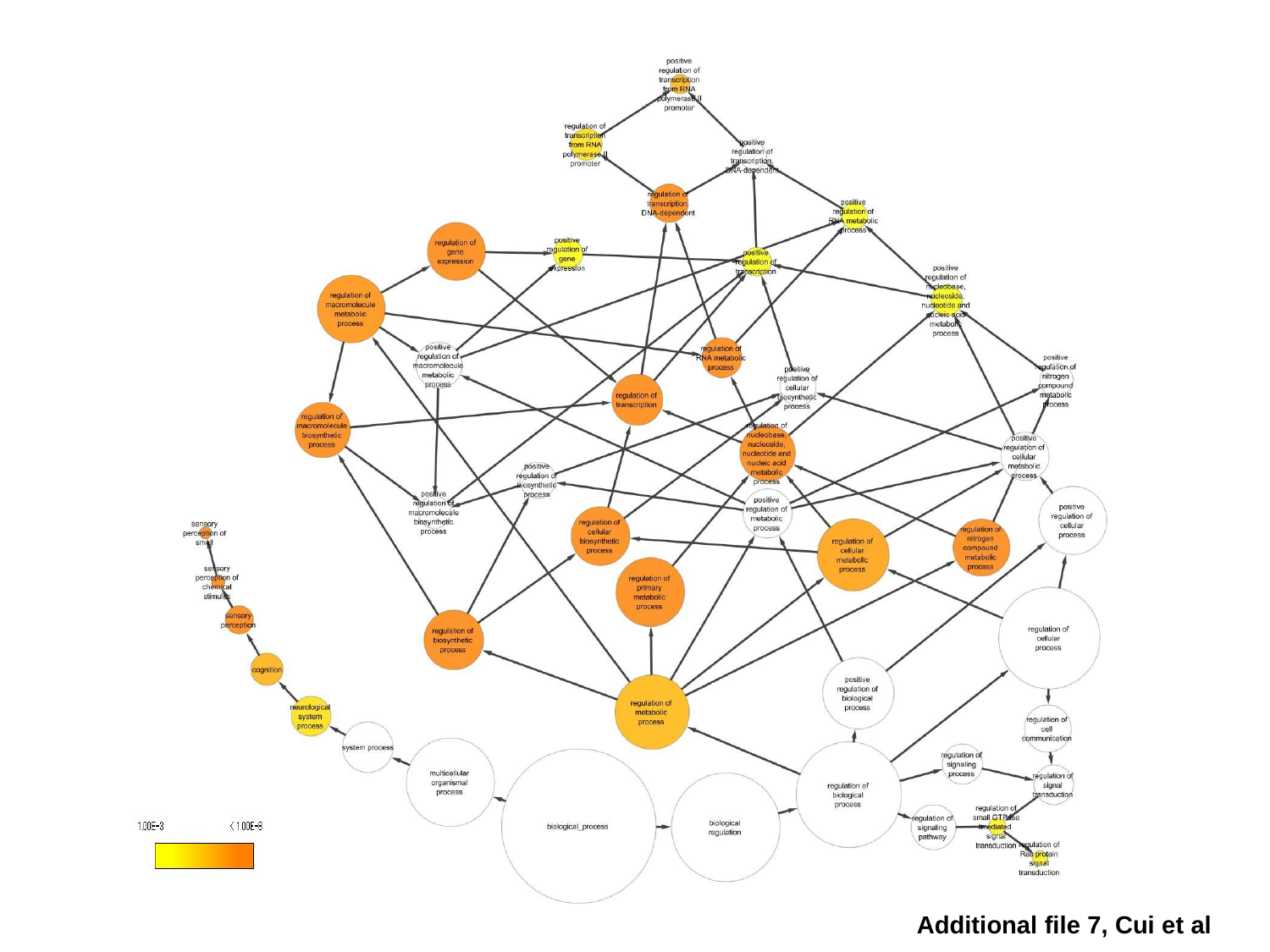

Additional file 7, Cui et al
