# Supplementary material for: Profiling and annotation of human kidney glomerulus proteome
Source: Proteome Sci. 2013 Apr 8;11:13. doi: 10.1186/1477-5956-11-13 (PMC3639854; doi:10.1186/1477-5956-11-13)
Supplement: Additional file 8 — Glomerular proteins matched in a KEGG “Regulation of Actin Cytoskeleton” pathway. All the identified proteins of the non-redundant, high-confidence dataset of glomerulus proteome consisting of 1,817 unique proteins representing 1,478 unique genes were analyzed by DAVID KEGG analysis. Matched proteins to components in the “Regulation of Actin Cytoskeleton” are indicated with asterisk. [file 1477-5956-11-13-S8.ppt]

## Slide 1
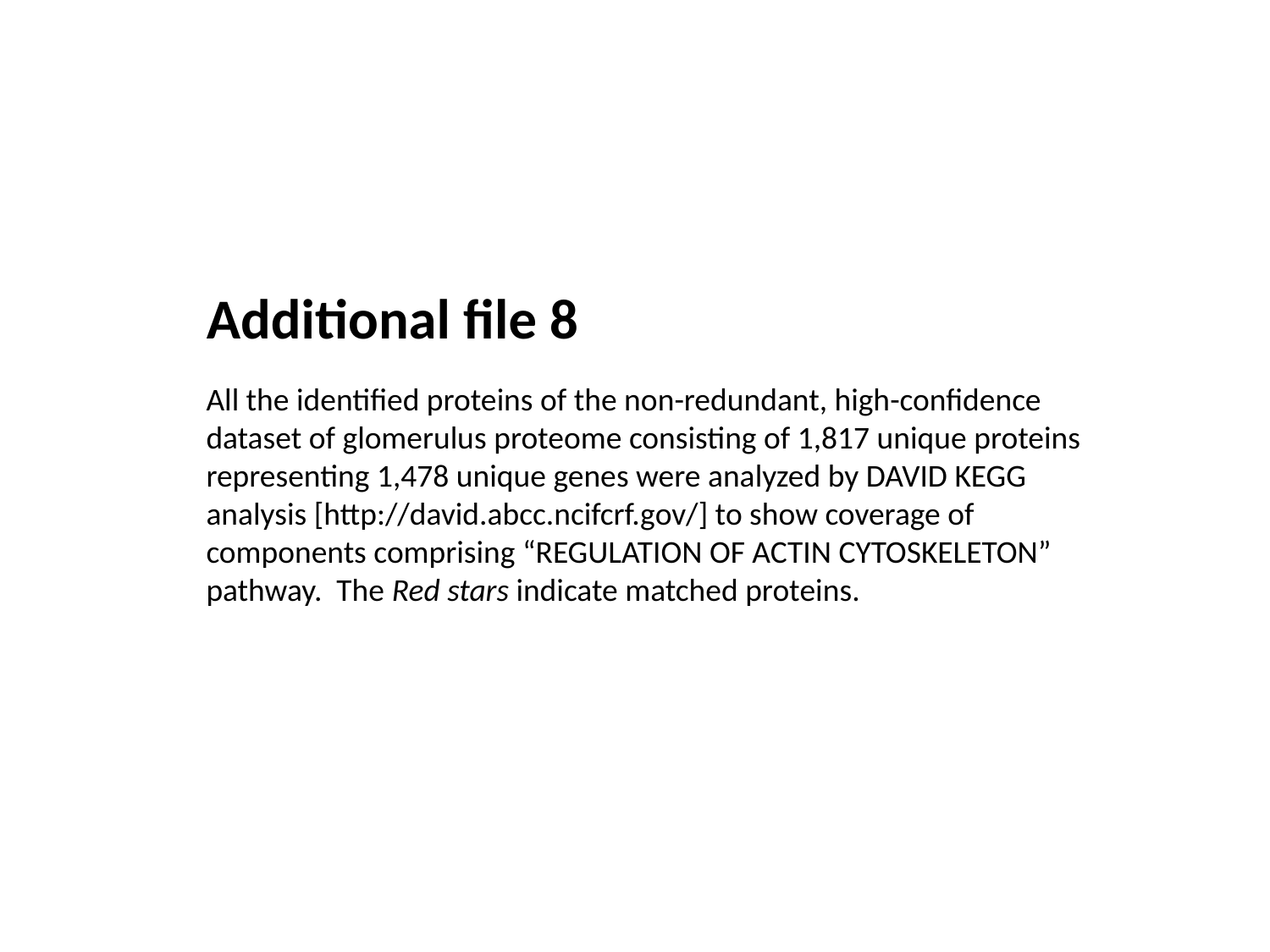

Additional file 8
All the identified proteins of the non-redundant, high-confidence dataset of glomerulus proteome consisting of 1,817 unique proteins representing 1,478 unique genes were analyzed by DAVID KEGG analysis [http://david.abcc.ncifcrf.gov/] to show coverage of components comprising “REGULATION OF ACTIN CYTOSKELETON” pathway. The Red stars indicate matched proteins.

## Slide 2
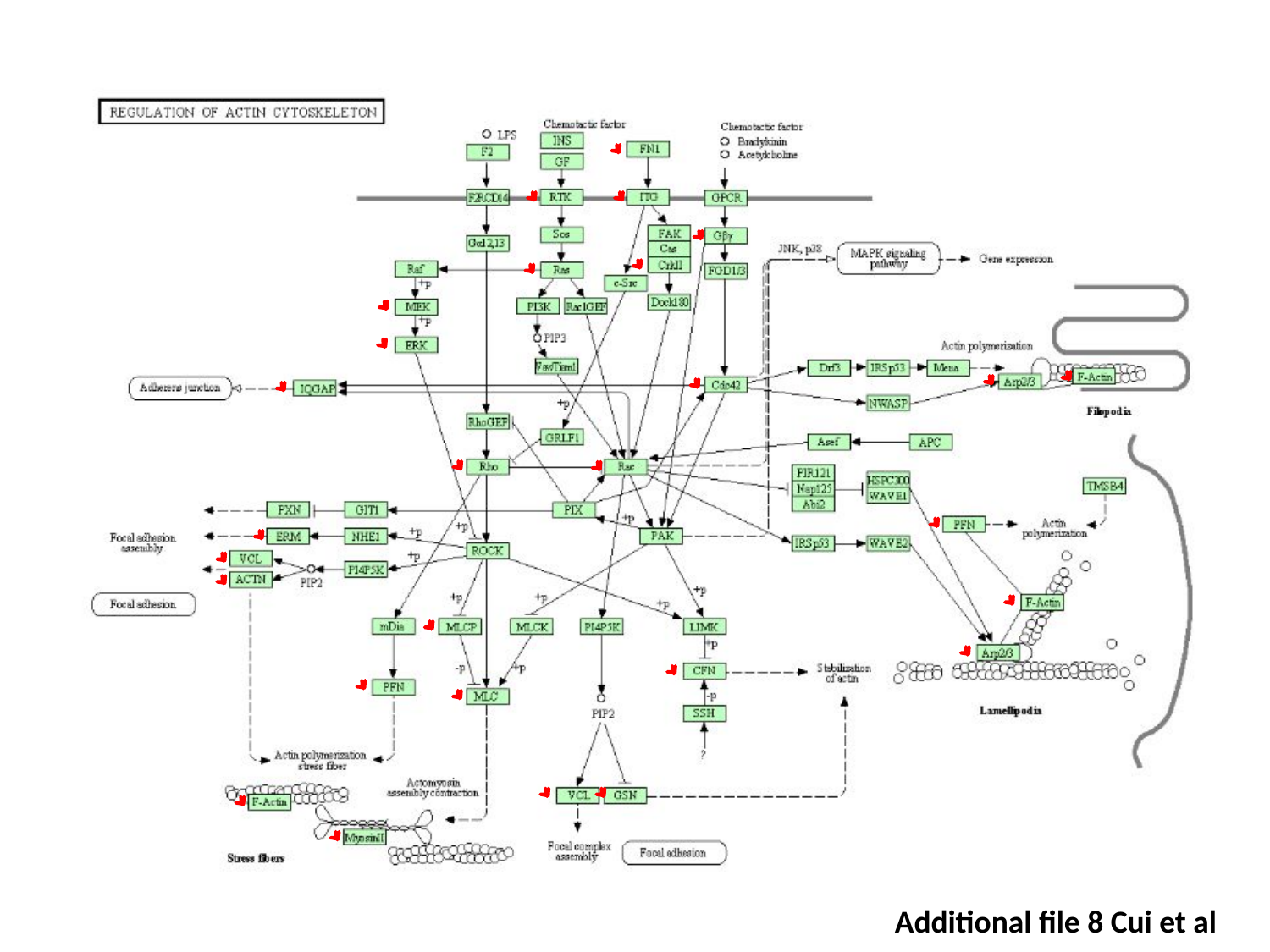

Additional file 8 Cui et al
