# Supplementary material for: Profiling and annotation of human kidney glomerulus proteome
Source: Proteome Sci. 2013 Apr 8;11:13. doi: 10.1186/1477-5956-11-13 (PMC3639854; doi:10.1186/1477-5956-11-13)
Supplement: Additional file 9 — An estimate of protein contamination from blood into glomerulus proteome by comparison of glomerulus proteome with plasma proteome. All the identified proteins of the non-redundant, high-confidence dataset of glomerulus proteome consisting of 1,817 unique proteins representing 1,478 unique genes were compared with the high-confidence, non-redundant dataset of normal human plasma proteome [States DJ et al., Nat. Biotech., 2006, 24, 333–338]. Among the 401 overlapping proteins, proteins annotated as “extracellular space” by GO Cellular Component vocabulary were selected as representing plasma proteins. The top 30 plasma proteins are listed in order according to the number of peptide matches. [file 1477-5956-11-13-S9.ppt]

## Slide 1
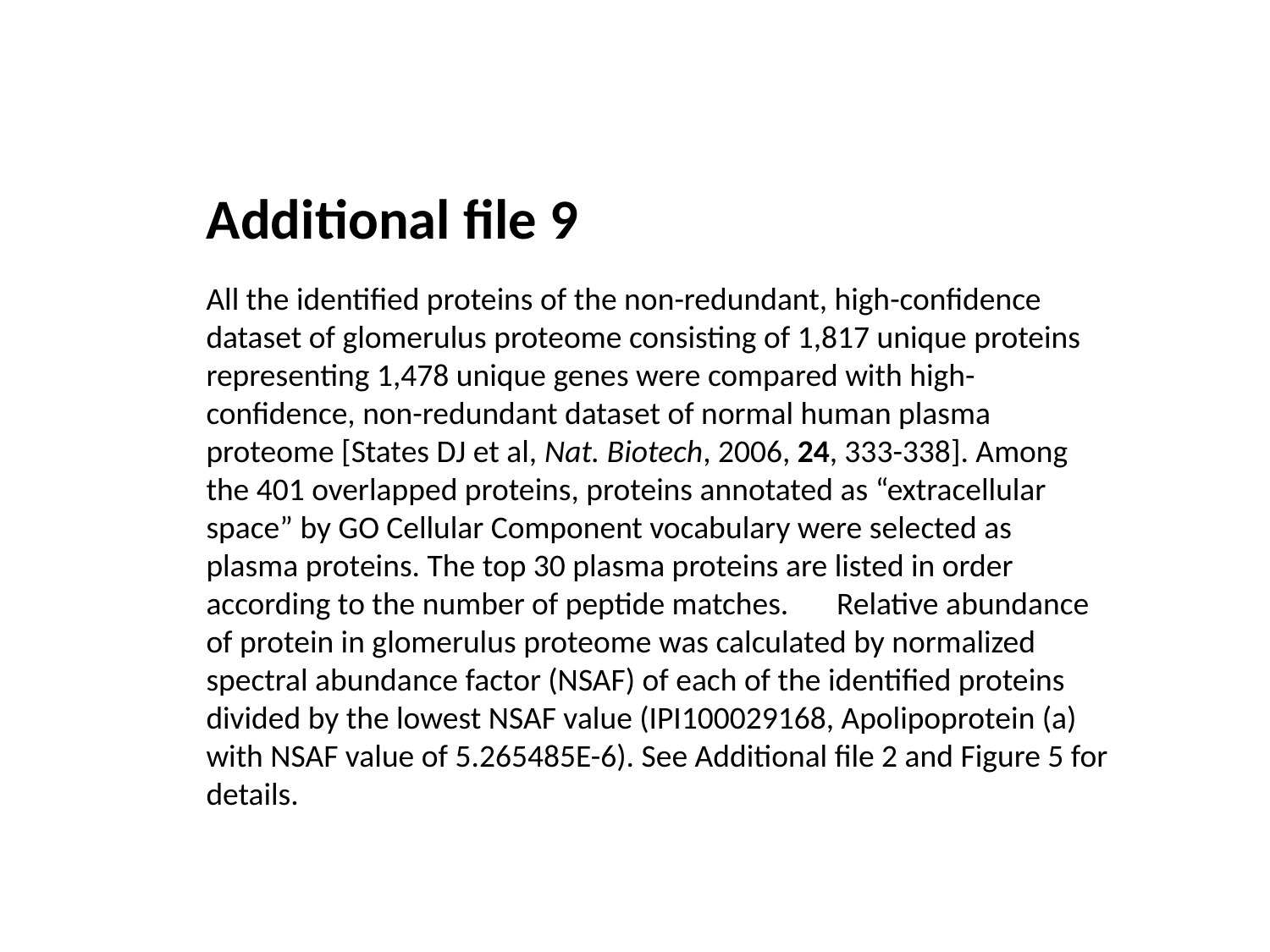

Additional file 9
All the identified proteins of the non-redundant, high-confidence dataset of glomerulus proteome consisting of 1,817 unique proteins representing 1,478 unique genes were compared with high-confidence, non-redundant dataset of normal human plasma proteome [States DJ et al, Nat. Biotech, 2006, 24, 333-338]. Among the 401 overlapped proteins, proteins annotated as “extracellular space” by GO Cellular Component vocabulary were selected as plasma proteins. The top 30 plasma proteins are listed in order according to the number of peptide matches.　Relative abundance of protein in glomerulus proteome was calculated by normalized spectral abundance factor (NSAF) of each of the identified proteins divided by the lowest NSAF value (IPI100029168, Apolipoprotein (a) with NSAF value of 5.265485E-6). See Additional file 2 and Figure 5 for details.

## Slide 2
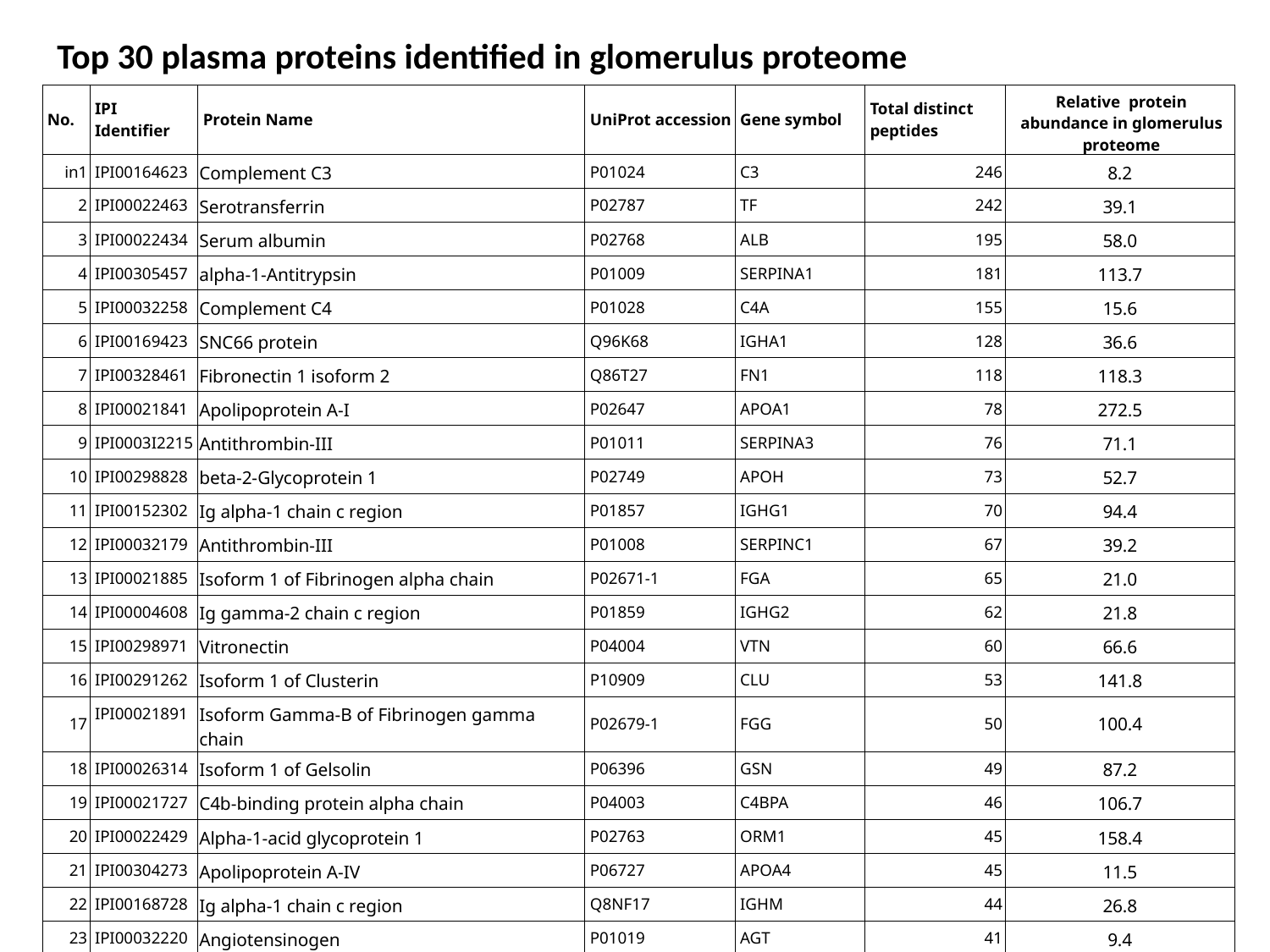

Top 30 plasma proteins identified in glomerulus proteome
| No. | IPI Identifier | Protein Name | UniProt accession | Gene symbol | Total distinct peptides | Relative protein abundance in glomerulus proteome |
| --- | --- | --- | --- | --- | --- | --- |
| in1 | IPI00164623 | Complement C3 | P01024 | C3 | 246 | 8.2 |
| 2 | IPI00022463 | Serotransferrin | P02787 | TF | 242 | 39.1 |
| 3 | IPI00022434 | Serum albumin | P02768 | ALB | 195 | 58.0 |
| 4 | IPI00305457 | alpha-1-Antitrypsin | P01009 | SERPINA1 | 181 | 113.7 |
| 5 | IPI00032258 | Complement C4 | P01028 | C4A | 155 | 15.6 |
| 6 | IPI00169423 | SNC66 protein | Q96K68 | IGHA1 | 128 | 36.6 |
| 7 | IPI00328461 | Fibronectin 1 isoform 2 | Q86T27 | FN1 | 118 | 118.3 |
| 8 | IPI00021841 | Apolipoprotein A-I | P02647 | APOA1 | 78 | 272.5 |
| 9 | IPI0003I2215 | Antithrombin-III | P01011 | SERPINA3 | 76 | 71.1 |
| 10 | IPI00298828 | beta-2-Glycoprotein 1 | P02749 | APOH | 73 | 52.7 |
| 11 | IPI00152302 | Ig alpha-1 chain c region | P01857 | IGHG1 | 70 | 94.4 |
| 12 | IPI00032179 | Antithrombin-III | P01008 | SERPINC1 | 67 | 39.2 |
| 13 | IPI00021885 | Isoform 1 of Fibrinogen alpha chain | P02671-1 | FGA | 65 | 21.0 |
| 14 | IPI00004608 | Ig gamma-2 chain c region | P01859 | IGHG2 | 62 | 21.8 |
| 15 | IPI00298971 | Vitronectin | P04004 | VTN | 60 | 66.6 |
| 16 | IPI00291262 | Isoform 1 of Clusterin | P10909 | CLU | 53 | 141.8 |
| 17 | IPI00021891 | Isoform Gamma-B of Fibrinogen gamma chain | P02679-1 | FGG | 50 | 100.4 |
| 18 | IPI00026314 | Isoform 1 of Gelsolin | P06396 | GSN | 49 | 87.2 |
| 19 | IPI00021727 | C4b-binding protein alpha chain | P04003 | C4BPA | 46 | 106.7 |
| 20 | IPI00022429 | Alpha-1-acid glycoprotein 1 | P02763 | ORM1 | 45 | 158.4 |
| 21 | IPI00304273 | Apolipoprotein A-IV | P06727 | APOA4 | 45 | 11.5 |
| 22 | IPI00168728 | Ig alpha-1 chain c region | Q8NF17 | IGHM | 44 | 26.8 |
| 23 | IPI00032220 | Angiotensinogen | P01019 | AGT | 41 | 9.4 |
| 24 | IPI00296099 | Thrombospondin-1 | P07996 | THBS1 | 40 | 11.7 |
| 25 | IPI00220327 | Keratin, type II cytoskeletal 1 | P04264 | KRT1 | 39 | 734.5 |
| 26 | IPI00020986 | Lumican | P51884 | LUM | 34 | 148.0 |
| 27 | IPI00022395 | Complement component C9 | P02748 | C9 | 33 | 8.1 |
| 28 | IPI00022426 | Protein AMBP | P02760 | AMBP | 33 | 25.8 |
| 29 | IPI00021842 | Apolipoprotein E | P02649 | APOE | 30 | 301.3 |
| 30 | IPI00294004 | Vitamin K-dependent protein S | P07225 | PROS1 | 29 | 6.7 |
