# Supplementary material for: Profiling and annotation of human kidney glomerulus proteome
Source: Proteome Sci. 2013 Apr 8;11:13. doi: 10.1186/1477-5956-11-13 (PMC3639854; doi:10.1186/1477-5956-11-13)
Supplement: Additional file 11 — The proteins that might be specifically or abundantly expressed in human glomerulus compared with mouse glomerulus. The human glomerular dataset was compared with the mouse glomerular dataset [Waanders et al., Proc. Natl. Acad. Sci. USA, 2009, 106, 18902–18907]. Gene symbols of the mouse dataset were converted to corresponding gene symbols of human ortholog genes by mapping them to an ortholog table of human and mouse genes based on an “Evola ortholog list” (version 7.5) created by the Human Invitational database (H-Inv DB). Proteins-corresponding genes uniquely identified in the human dataset were examined for their cellular localization by searching in the Human Protein Atlas (version 9.0). [file 1477-5956-11-13-S11.ppt]

## Slide 1
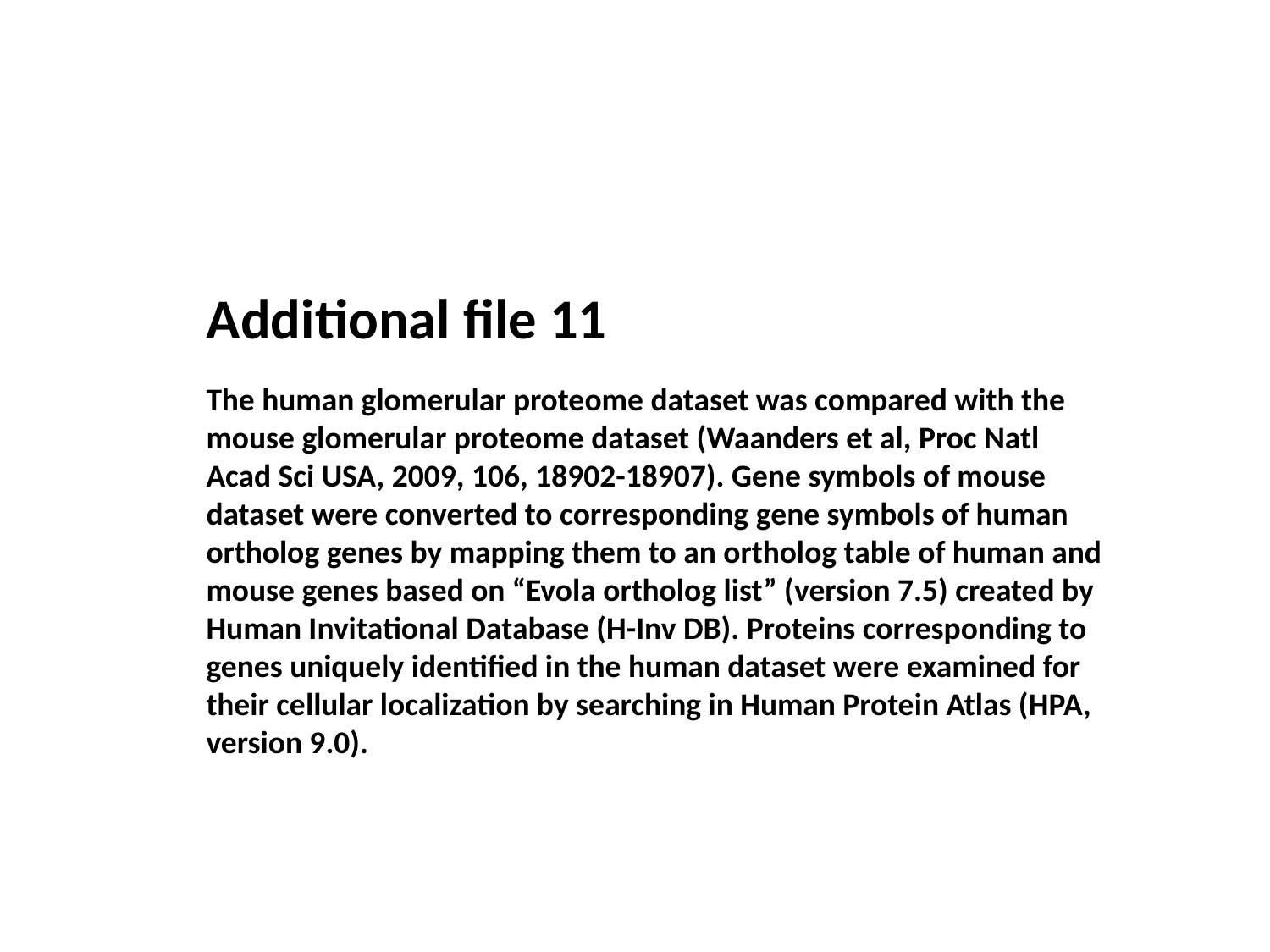

Additional file 11
The human glomerular proteome dataset was compared with the mouse glomerular proteome dataset (Waanders et al, Proc Natl Acad Sci USA, 2009, 106, 18902-18907). Gene symbols of mouse dataset were converted to corresponding gene symbols of human ortholog genes by mapping them to an ortholog table of human and mouse genes based on “Evola ortholog list” (version 7.5) created by Human Invitational Database (H-Inv DB). Proteins corresponding to genes uniquely identified in the human dataset were examined for their cellular localization by searching in Human Protein Atlas (HPA, version 9.0).

## Slide 2
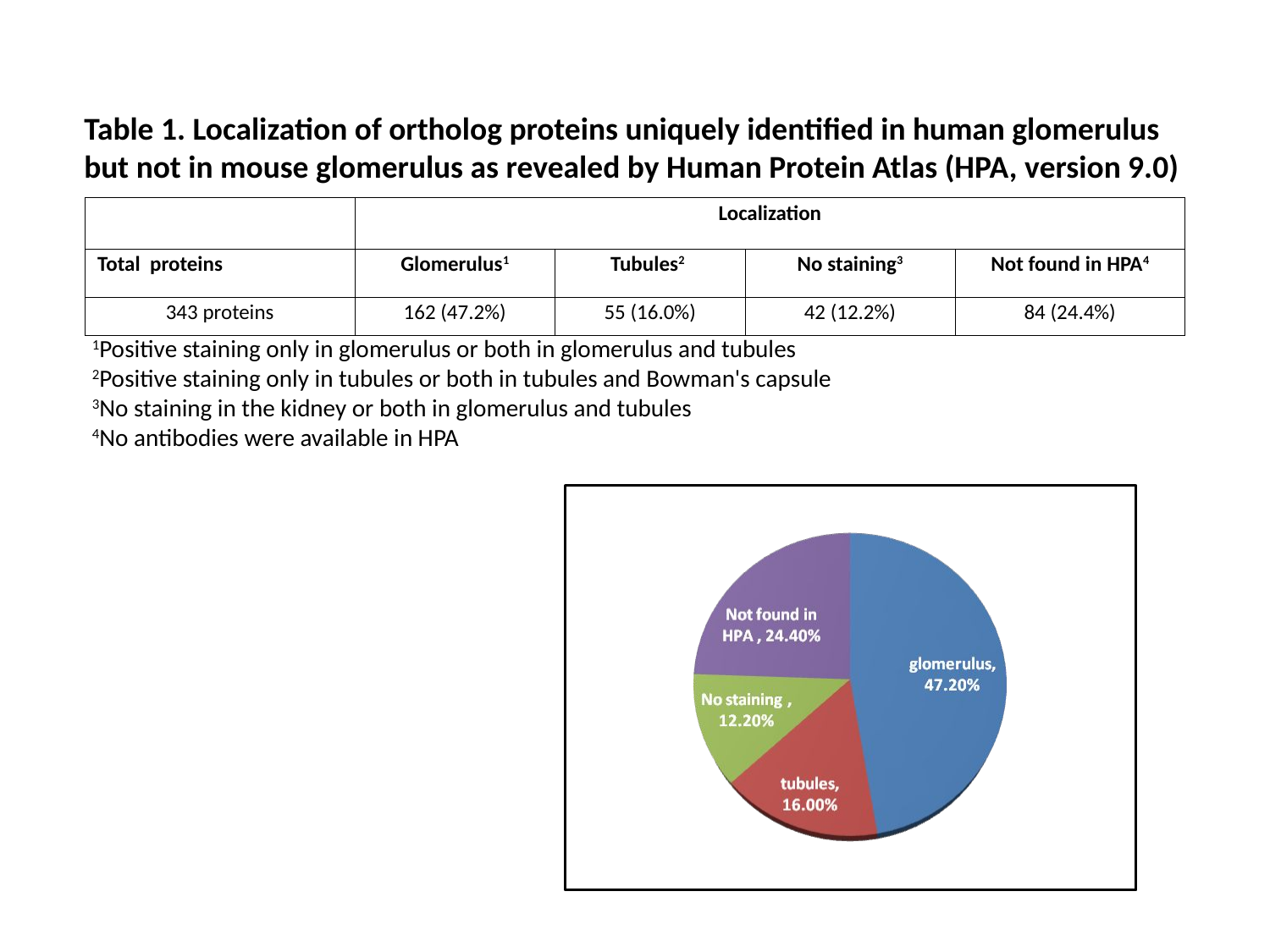

Table 1. Localization of ortholog proteins uniquely identified in human glomerulus but not in mouse glomerulus as revealed by Human Protein Atlas (HPA, version 9.0)
| | Localization | | | |
| --- | --- | --- | --- | --- |
| Total proteins | Glomerulus1 | Tubules2 | No staining3 | Not found in HPA4 |
| 343 proteins | 162 (47.2%) | 55 (16.0%) | 42 (12.2%) | 84 (24.4%) |
| |
| --- |
| |
| --- |
1Positive staining only in glomerulus or both in glomerulus and tubules
2Positive staining only in tubules or both in tubules and Bowman's capsule
3No staining in the kidney or both in glomerulus and tubules
4No antibodies were available in HPA

## Slide 3
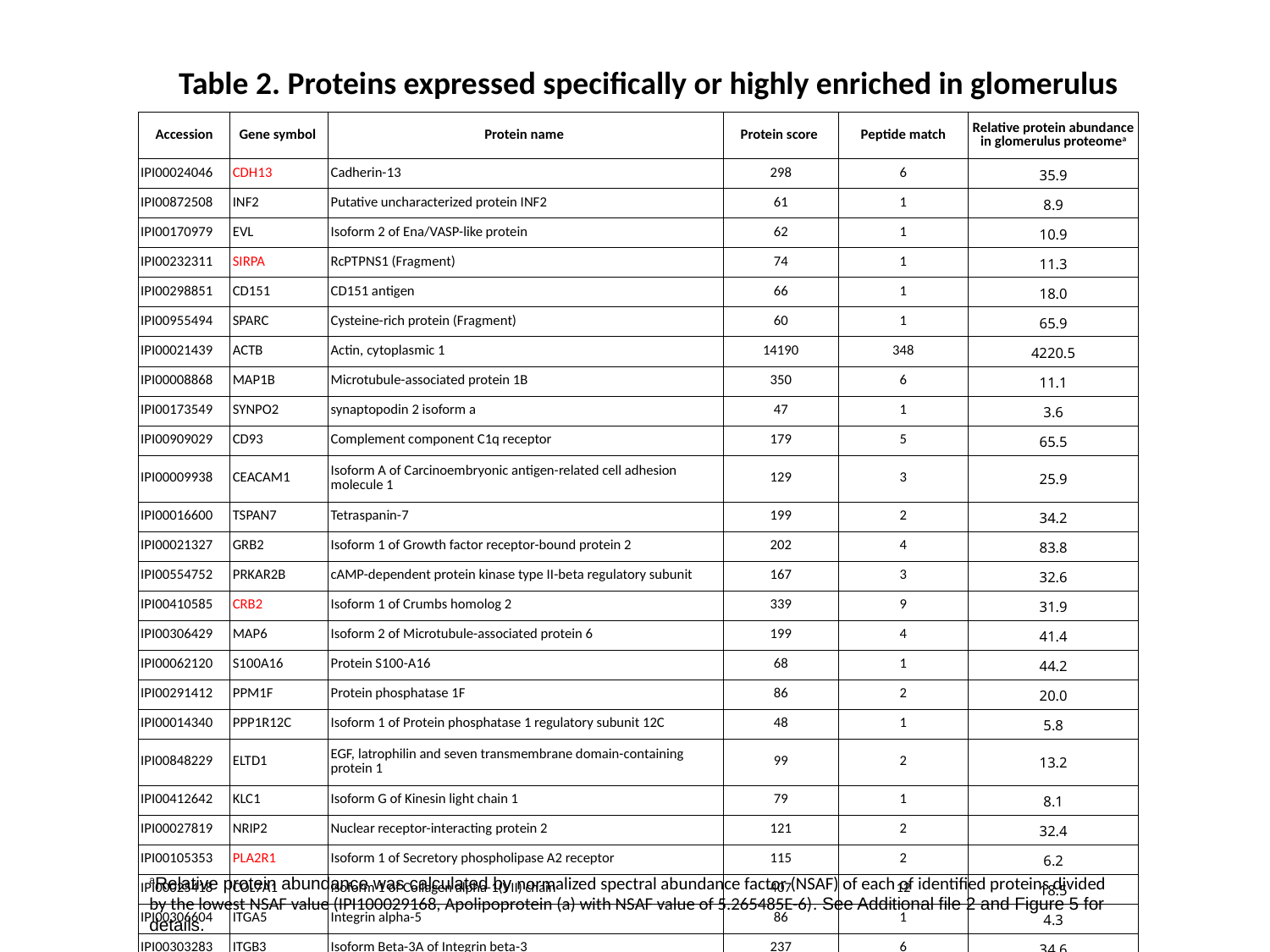

Table 2. Proteins expressed specifically or highly enriched in glomerulus
| |
| --- |
| Accession | Gene symbol | Protein name | Protein score | Peptide match | Relative protein abundance in glomerulus proteomea |
| --- | --- | --- | --- | --- | --- |
| IPI00024046 | CDH13 | Cadherin-13 | 298 | 6 | 35.9 |
| IPI00872508 | INF2 | Putative uncharacterized protein INF2 | 61 | 1 | 8.9 |
| IPI00170979 | EVL | Isoform 2 of Ena/VASP-like protein | 62 | 1 | 10.9 |
| IPI00232311 | SIRPA | RcPTPNS1 (Fragment) | 74 | 1 | 11.3 |
| IPI00298851 | CD151 | CD151 antigen | 66 | 1 | 18.0 |
| IPI00955494 | SPARC | Cysteine-rich protein (Fragment) | 60 | 1 | 65.9 |
| IPI00021439 | ACTB | Actin, cytoplasmic 1 | 14190 | 348 | 4220.5 |
| IPI00008868 | MAP1B | Microtubule-associated protein 1B | 350 | 6 | 11.1 |
| IPI00173549 | SYNPO2 | synaptopodin 2 isoform a | 47 | 1 | 3.6 |
| IPI00909029 | CD93 | Complement component C1q receptor | 179 | 5 | 65.5 |
| IPI00009938 | CEACAM1 | Isoform A of Carcinoembryonic antigen-related cell adhesion molecule 1 | 129 | 3 | 25.9 |
| IPI00016600 | TSPAN7 | Tetraspanin-7 | 199 | 2 | 34.2 |
| IPI00021327 | GRB2 | Isoform 1 of Growth factor receptor-bound protein 2 | 202 | 4 | 83.8 |
| IPI00554752 | PRKAR2B | cAMP-dependent protein kinase type II-beta regulatory subunit | 167 | 3 | 32.6 |
| IPI00410585 | CRB2 | Isoform 1 of Crumbs homolog 2 | 339 | 9 | 31.9 |
| IPI00306429 | MAP6 | Isoform 2 of Microtubule-associated protein 6 | 199 | 4 | 41.4 |
| IPI00062120 | S100A16 | Protein S100-A16 | 68 | 1 | 44.2 |
| IPI00291412 | PPM1F | Protein phosphatase 1F | 86 | 2 | 20.0 |
| IPI00014340 | PPP1R12C | Isoform 1 of Protein phosphatase 1 regulatory subunit 12C | 48 | 1 | 5.8 |
| IPI00848229 | ELTD1 | EGF, latrophilin and seven transmembrane domain-containing protein 1 | 99 | 2 | 13.2 |
| IPI00412642 | KLC1 | Isoform G of Kinesin light chain 1 | 79 | 1 | 8.1 |
| IPI00027819 | NRIP2 | Nuclear receptor-interacting protein 2 | 121 | 2 | 32.4 |
| IPI00105353 | PLA2R1 | Isoform 1 of Secretory phospholipase A2 receptor | 115 | 2 | 6.2 |
| IPI00025418 | COL7A1 | Isoform 1 of Collagen alpha-1(VII) chain | 407 | 12 | 18.5 |
| IPI00306604 | ITGA5 | Integrin alpha-5 | 86 | 1 | 4.3 |
| IPI00303283 | ITGB3 | Isoform Beta-3A of Integrin beta-3 | 237 | 6 | 34.6 |
| IPI00026337 | RANBP3 | Isoform 1 of Ran-binding protein 3 | 181 | 2 | 16.0 |
| IPI00432337 | ERLEC1 | Isoform 2 of Endoplasmic reticulum lectin 1 | 203 | 4 | 42.4 |
| IPI00293336 | MBLAC2 | Isoform 1 of Metallo-beta-lactamase domain-containing protein 2 | 309 | 8 | 130.4 |
| IPI00032409 | ROBLD3 | Isoform 1 of Mitogen-activated protein-binding protein-interacting protein | 167 | 3 | 109.2 |
aRelative protein abundance was calculated by normalized spectral abundance factor (NSAF) of each of identified proteins divided by the lowest NSAF value (IPI100029168, Apolipoprotein (a) with NSAF value of 5.265485E-6). See Additional file 2 and Figure 5 for details.
